# Application of transfer learning for rapid calibration of spatially resolved diffuse reflectance probes for extraction of tissue optical properties

**DOI:** 10.1117/1.JBO.29.2.027004

**Published:** 2024-02-28

**Authors:** Md Nafiz Hannan, Timothy M. Baran

**Affiliations:** a University of Rochester, Department of Physics and Astronomy, Rochester, New York, United States; b University of Rochester Medical Center, Department of Imaging Sciences, Rochester, New York, United States; c University of Rochester, Department of Biomedical Engineering, Rochester, New York, United States; d University of Rochester, The Institute of Optics, Rochester, New York, United States

**Keywords:** diffuse reflectance spectroscopy, machine learning, neural network, transfer learning, Monte Carlo simulation

## Abstract

**Significance:**

Treatment planning for light-based therapies including photodynamic therapy requires tissue optical property knowledge. This is recoverable with spatially resolved diffuse reflectance spectroscopy (DRS) but requires precise source–detector separation (SDS) determination and time-consuming simulations.

**Aim:**

An artificial neural network (ANN) to map from DRS at multiple SDS to optical properties was created. This trained ANN was adapted to fiber-optic probes with varying SDS using transfer learning (TL).

**Approach:**

An ANN mapping from measurements to Monte Carlo simulation to optical properties was created with one fiber-optic probe. A second probe with different SDS was used for TL algorithm creation. Data from a third were used to test this algorithm.

**Results:**

The initial ANN recovered absorber concentration with RMSE=0.29  μM (7.5% mean error) and $\mu_{s}^{\prime }$ at 665 nm ($\mu_{s,665}^{\prime }$) with $\mathrm{RMSE}=0.77\;{\mathrm{cm}}^{-1}$ (2.5% mean error). For probe 2, TL significantly improved absorber concentration (0.38 versus 1.67  μM RMSE, p=0.0005) and ${\mu^{\prime }}_{s,665}$ (0.71 versus $1.8\;{\mathrm{cm}}^{-1}$ RMSE, p=0.0005) recovery. A third probe also showed improved absorber (0.7 versus 4.1  μM RMSE, p<0.0001) and $\mu_{s,665}^{\prime }$ (1.68 versus $2.08\;{\mathrm{cm}}^{-1}$ RMSE, p=0.2) recovery.

**Conclusions:**

TL-based probe-to-probe calibration can rapidly adapt an ANN created for one probe to similar target probes, enabling accurate optical property recovery with the target probe.

## 1 Introduction

Photodynamic therapy (PDT), which produces an antimicrobial effect through the photochemical production of reactive oxygen species, is a promising treatment modality for eradication of infectious disease.^1^ We are particularly interested in the use of PDT to treat deep tissue abscesses, which are localized collections of infected, purulent fluid surrounded by a fibrous pseudocapsule. We have shown that PDT is safe and feasible for this purpose in a phase 1 clinical trial,^2^ and that PDT is efficacious against bacteria cultured from human abscesses.^3^ Effective PDT requires sufficient doses of both the photosensitizer and treatment light. As the light dose is dependent upon the optical properties of the target tissue, namely absorption and scattering, accurate measurement of tissue optical properties is required for designing efficacious PDT treatment plans. To that end, we have constructed and validated a spatially resolved diffuse reflectance spectroscopy (DRS) system that is capable of extracting tissue optical properties using a fiber-optic probe placed in contact with the abscess wall.^4^ Critically, the fiber-optic probe used to deliver light and collect diffusely reflected light was required to fit through the drainage catheter used for standard of care abscess drainage, which has an inner diameter of only 2 mm. This spectroscopy system was utilized during our recently completed phase 1 clinical trial^2^ to quantify human abscess wall optical properties.^5^ Substantial interpatient variability in the abscess wall optical properties was found.^5^ We have shown that patient-specific treatment planning incorporating knowledge of these optical properties greatly improves delivery of an efficacious fluence rate to the abscess wall.^6^^,^^7^ Measurement of individual patient optical properties is therefore of high importance for the clinical success of PDT for this application.

To extract optical properties from DRS, different approaches have been used, broadly categorized into two groups: analytical and numerical. Analytical approximations to the radiative transport equation, such as the diffusion^8^^,^^9^ and $P_{3}$^10^ approximations have been utilized by many research groups. However, these approximations place limitations on source–detector separation (SDS), requiring detectors to be multiple transport mean free paths (mfp′) from the source, where ${\mathrm{mfp}}^{\prime }=1/(\mu_{a}+\mu_{s}^{\prime })$ ($\mu_{a}$ = absorption coefficient, $\mu_{s}^{\prime }$ = reduced scattering coefficient). We categorized SDS as “short” for distances less than one mfp′ for typical tissue optical properties ($\mu_{a}=0.2\;{\mathrm{cm}}^{-1}$, $\mu_{s}^{\prime }=10\;{\mathrm{cm}}^{-1}$, ${\mathrm{mfp}}^{\prime }=0.98\;\mathrm{mm}$). As described above, our primary clinical target requires that a fiber-optic probe with multiple SDS must fit through a catheter with a diameter of 2 mm, limiting the majority of our SDS to be less than one mfp′. As a consequence, these analytical approximations are not valid for the probes we aim to clinically deploy and more broadly for spatially resolved diffuse reflectance at short SDS. Many research groups have, therefore, employed numerical approaches, such as the Monte Carlo lookup table (MCLUT) inverse model for optical property recovery.^4^^,^^11^ This approach is more flexible in terms of SDS range and is also valid for a wide range of optical properties. However, the usage of MC requires accurate SDS measurements for model creation and generation of an extensive MCLUT, which remains resource and time-intensive despite graphics processing unit (GPU) acceleration. Moreover, a distinct MC library must be generated for every probe employed. This is undesirable for cases where similar but distinct probes need be deployed, such as for single-use devices.

With advancements in computational power and machine learning (ML) algorithms, numerous research groups have turned to fully data-driven ML approaches for recovering optical properties from DRS. Farrell et al.^8^ pioneered the use of an artificial neural network (ANN) trained with the diffusion approximation to extract optical properties from simulated experimental data. Recognizing the limitations of the diffusion approximation, Kienle et al.^12^ subsequently employed an ANN trained with MC simulations. More recently, Nguyen et al.^13^ demonstrated that deep learning models showed lower error and faster runtime compared to a MCLUT inverse model. Lan et al.^14^ developed an ANN model that achieved comparable accuracy to the traditional Monte Carlo-based inverse model while offering improved speed and flexibility. While these studies used ANNs trained on simulated data, Pfefer et al.^15^ also examined the case of an end-to-end network for direct prediction of optical properties from phantom measurements. However, this approach required a large database of phantom data for training.

These prior studies were confined to the performance of ANN-based optical property recovery for a single device. Data obtained from analytical solutions^8^ and/or MC libraries^12^^,^^14^ for a specific probe in combination with phantom data collected by that probe were used to train these ANN models. None of these studies showed extension of ANNs to the case of unseen probes with variable SDS. As the ANNs created by these groups were probe-specific, they are not suitable for clinical adoption or more widespread application where rapid calibration of a large number of new devices is required. An ANN created for one specific device cannot be expected to perform well for other probes having dissimilar SDS, as the DRS signal will vary in unknown ways. To circumvent this with current methods, either the probe SDS would need to be very tightly controlled or a separate ANN would need to be created for each probe. Both of these solutions would impede clinical implementation by increasing costs due to tight tolerancing and prolonging the time required for calibration.

To facilitate our envisioned multicenter clinical trials and eventual widespread clinical adaptation, we propose a transfer learning (TL) approach for rapid probe-to-probe calibration. TL is a widely employed approach in the ML field, wherein models trained for one specific task are partially retrained to address a related task that has limited available data. We hypothesize that using a feature-extraction based TL algorithm, an ANN created for one probe can be adapted to similar probes via a small dataset consisting of only a few calibration spectra collected by the target probe. This will enable the creation of an initial ANN for a single probe, which can be easily adapted to target probes without the need for the time and resource-intensive steps mentioned earlier, thus rendering large-scale clinical adoption more feasible.

This study followed a three-step workflow. First, an initial ANN was created and validated using data captured with a single-fiber-optic probe (probe 1). This initial ANN served as a pretrained model for subsequent TL steps. Second, we utilized data from a second fiber-optic probe with slight differences in SDS (probe 2) to create and validate a TL algorithm, which included selecting and optimizing the TL algorithm, TL dataset size, and associated hyperparameters. Finally, this TL algorithm was applied to data from a third fiber optic probe (probe 3) in a “real-world” scenario to independently assess its performance and applicability.

## 2 Methods

### 2.1 Spectroscopy System

The fiber-optic probe-based diffuse reflectance system utilized in this study has been extensively described in our earlier publication.^4^ Briefly, it is a continuous wave spectroscopy system in which broadband light from a tungsten halogen lamp (HL-2000-HP-FHSA, Ocean Optics, Inc., Largo, Florida, United States) is emitted from a source fiber into the sample. Eight detector fibers, which are placed at different SDSs from the source fiber on the face of the probe, collect the reflected light for detection by a spectrometer (QE Pro, Ocean Optics). An encrypted, password-protected laptop computer is used to operate this system via a custom interface created in LabVIEW (National Instruments, Austin, Texas, United Sates).

### 2.2 Probe Characterization

Three custom-made fiber optic probes were used, each having an outer diameter of 2 mm. These probes were manufactured by Pioneer Optics Company (Bloomfield, Connecticut, United States), following the same design specifications. These specifications involved positioning the fiber faces at the distal end of the probe in a “plus” pattern, with a desired distance of 300  μm between adjacent fiber centers. While the design intended for each of these probes to possess the same SDS, the delivered probes had varying SDS values compared to each other. At the short separations used in these probes, precise knowledge of SDS is important for accurate recovery of tissue optical properties.

**Fig. 1:**
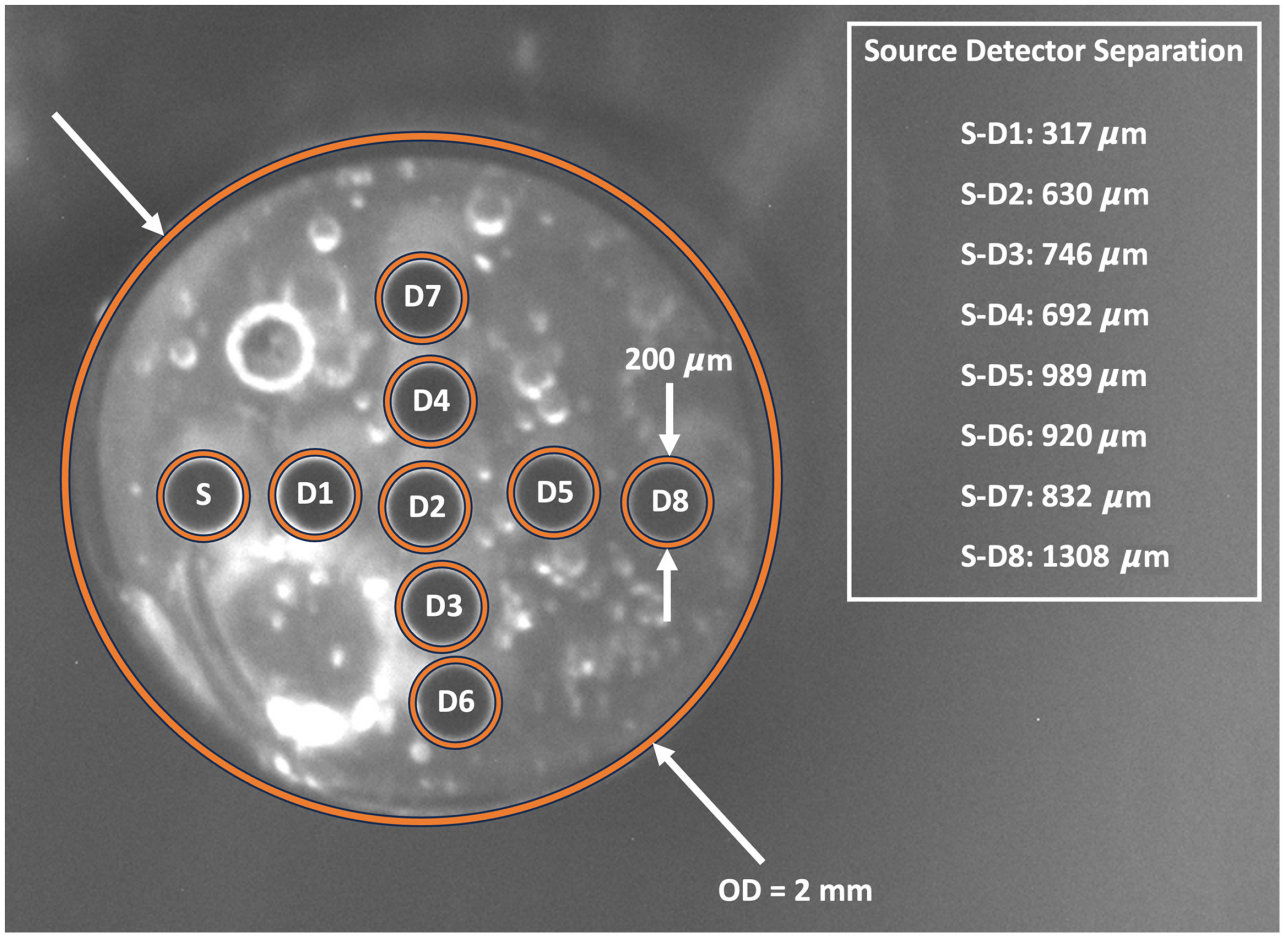
Image of the distal face of probe 1 showing the 200  μm diameter fibers. The source fiber (S) and detector fibers (D1 to D8) are denoted using orange circles. The outside diameter is abbreviated as “OD.”

In order to determine the actual SDS for each probe, a stereomicroscope (SMZ1500, Nikon Instruments, Melville, New York, United States) was used. The distal end of each probe was imaged both with and without a US Air Force resolution target (USAF 1951 1X, Edmund Optics, Inc., Barrington, New Jersey, United States) in the same frame. The air force target was used as a distance reference. The centers of each fiber were identified and SDS for each fiber were determined. An image of the distal face of probe 1 showing the position of the source and detector fibers is presented in Fig. 1. The SDS for all eight detectors fibers for each probe is given in Table 1.

**Table 1:** SDS in μm for the eight detector fibers of probes 1, 2, and 3.

Probe no.	Detector fiber 1	Detector fiber 2	Detector fiber 3	Detector fiber 4	Detector fiber 5	Detector fiber 6	Detector fiber 7	Detector fiber 8
Probe 1	317	630	746	692	989	920	832	1308
Probe 2	315	648	738	761	985	914	911	1342
Probe 3	325	736	804	829	1082	951	990	1424

### 2.3 Creation and Validation of Initial ANN (${\mathrm{ANN}}_{1}$)

**Fig. 2:**
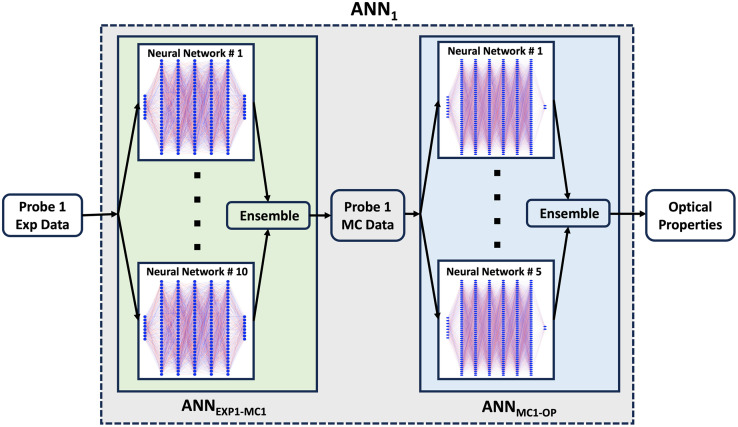
Initial ANN (${\mathrm{ANN}}_{1}$) for optical property extraction, which was created by combining ${\mathrm{ANN}}_{\mathrm{EXP}1\text{-}\mathrm{MC}1}$ ensemble and ${\mathrm{ANN}}_{\mathrm{MC}1\text{-}\mathrm{OP}}$ ensemble.

An ANN approach was employed to recover optical properties from diffuse reflectance spectra (DRS). An initial ANN (${\mathrm{ANN}}_{1}$) was trained to map from the experimentally collected DRS for probe 1 to corresponding optical properties (absorption and reduced scattering spectra). It had eight input nodes, one for each SDS, and two output nodes, one for each optical property (absorption and reduced scattering). It was created by combining two separate ANN ensembles, illustrated in Fig. 2. The first ANN ensemble (${\mathrm{ANN}}_{\mathrm{EXP}1\text{-}\mathrm{MC}1}$) takes in experimental diffuse reflectance intensities at the eight detector fibers as inputs, with outputs corresponding to Monte Carlo simulated reflectance intensities for these eight fibers. The second ANN ensemble (${\mathrm{ANN}}_{\mathrm{MC}1\text{-}\mathrm{OP}}$) takes in these eight simulated reflectance intensities and outputs corresponding optical properties. To get the final output of the ensemble models, predictions of individual ANNs were averaged. ANNs used in this research were created using the Keras API.^16^

#### 2.3.1 Creation and validation of ${\mathrm{ANN}}_{\mathrm{EXP}1\text{-}\mathrm{MC}1}$ network

The ${\mathrm{ANN}}_{\mathrm{EXP}1\text{-}\mathrm{MC}1}$ network was an ensemble of 10 individual ANNs as shown in Fig. 2. To balance between training time and optimized TL performance with a limited amount of data, we opted for an ensemble approach with 10 individual ANNs for the ${\mathrm{ANN}}_{\mathrm{EXP}\text{-}\mathrm{MC}1}$ network (see Fig. S3 in the Supplementary Material for more details). As the goal was to enable rapid TL on this ${\mathrm{ANN}}_{\mathrm{EXP}1\text{-}\mathrm{MC}1}$ network with a limited dataset, a simpler network structure was utilized than that described below for ${\mathrm{ANN}}_{\mathrm{MC}1\text{-}\mathrm{OP}}$.

To train the individual ANNs of the ${\mathrm{ANN}}_{\mathrm{EXP}1\text{-}\mathrm{MC}1}$ network, an 80% train, 20% validation split was performed on the ${\mathrm{TV}}_{\mathrm{EXP}1\text{-}\mathrm{MC}1}$ dataset described in the section “Probe 1 dataset,” with different random initializations for each ANN. Weight initialization used the Glorot uniform initialization method.^17^ Experimental reflectance spectra were treated as arrays of independent data points, with no knowledge of wavelength entering training. Training and validation data were randomly sampled from this concatenated, wavelength-independent dataset. The input signals (experimental reflectance intensity) were not scaled. However, as the outputs (simulated reflectance intensity) of different fibers were of vastly different magnitudes, they were rescaled to [0, 1] range using min–max normalization. Each individual ANN was comprised of an input layer with 8 fully connected neurons (one neuron for each SDS), 5 hidden layers with 30 fully connected neurons in each layer, along an output layer comprising 8 fully connected neurons, resulting in a total of 4238 trainable parameters for each ANN. This optimal number of hidden layers and number of neurons in these layers was found using KerasTuner,^16^ and the details of which are provided in Fig. S1 in the Supplementary Material. The hidden layers used rectified linear unit (ReLU) activation, and the output layer employed a linear activation function. Training was continued for up to 1000 epochs. An early stopping callback was implemented to halt training if there was no improvement in the validation loss for 50 epochs, preventing overfitting. The model that demonstrated the best performance on the validation set during training was restored and saved. The Adam optimizer with a mean-squared-error (MSE) cost function was used with a learning rate of 0.0005.

#### 2.3.2 Creation and validation of ${\mathrm{ANN}}_{\mathrm{MC}1\text{-}\mathrm{OP}}$ network

The optimal ${\mathrm{ANN}}_{\mathrm{MC}1\text{-}\mathrm{OP}}$ network was an ensemble of 5 ANNs, each with 6 hidden layers as depicted in Fig. 2. The details of the optimization are provided in Fig. S2 in the Supplementary Material. The ${\mathrm{TV}}_{\mathrm{MC}1\text{-}\mathrm{OP}1}$ dataset described in Sec. 2.5.2 was used to train this network. Both the input and output data were scaled to [0, 1] range during training, using the known range of simulated data. During inference, the outputs of the network were scaled back to the original, absolute range using inverse scaling to obtain absolute values of optical properties. The dataset was randomly divided into training set (80%) and test set (20%). An automated algorithm called deep jointly informed neural networks (DJINN)^18^ was used for automatic selection of the best ANN structure and hyperparameters without the need for time-consuming manual searching. DJINN automatically determined the near-optimal hyperparameters for our dataset, which were 250 epochs, batch size of 4916, and a learning rate of 0.007. By automating hyperparameter tuning and network structure selection, DJINN enabled us to obtain a high-performance model more efficiently. DJINN employed the Adam optimizer to minimize the MSE loss function. Each hidden layer utilized the ReLU activation function, and linear activation was applied to the output layers.

Finally, ${\mathrm{ANN}}_{\mathrm{EXP}1\text{-}\mathrm{MC}1}$ and ${\mathrm{ANN}}_{\mathrm{MC}1\text{-}\mathrm{OP}}$ were integrated end-to-end to form the initial ANN (${\mathrm{ANN}}_{1}$) as illustrated in Fig. 2. To independently assess its performance, we subjected ${\mathrm{ANN}}_{1}$ to evaluation using a distinct test dataset ($E_{\mathrm{EXP}1\text{-}\mathrm{OP}1}$) that was not included in training or validation. The performance of ${\mathrm{ANN}}_{1}$ was evaluated based on its ability to accurately predict both absorption ($\mu_{a}$) and reduced scattering ($\mu_{s}^{\prime }$) coefficients. Predicted absorption spectra were fitted with the known chromophore basis function to retrieve absorber concentration. Predicted reduced scattering coefficient was fitted with a power-law relationship of the form $\mu_{s}^{\prime }=a{\left(\lambda /\lambda_{0}\right)}^{-b}$, where a and b are the fitting coefficients, λ corresponds to wavelength in nm, and $\lambda_{0}$ is a normalization wavelength. This ${\mathrm{ANN}}_{1}$ served as the basis for the TL technique discussed in the next section.

### 2.4 Transfer Learning

In this study, a feature extraction technique was used for TL. For this technique, a pretrained model’s intermediate layers are used as feature extractors to capture relevant patterns and representations from data, followed by training additional task-specific layers on top to adapt the model for a specific task. To achieve this, only the output layer or the top few layers of the pretrained model are made trainable while all other layers remain frozen. By training these unfrozen layers using a small TL dataset, the pretrained model is adapted to the target task. This approach is most suitable when the target dataset is similar to the data the model was pretrained on.

**Fig. 3:**
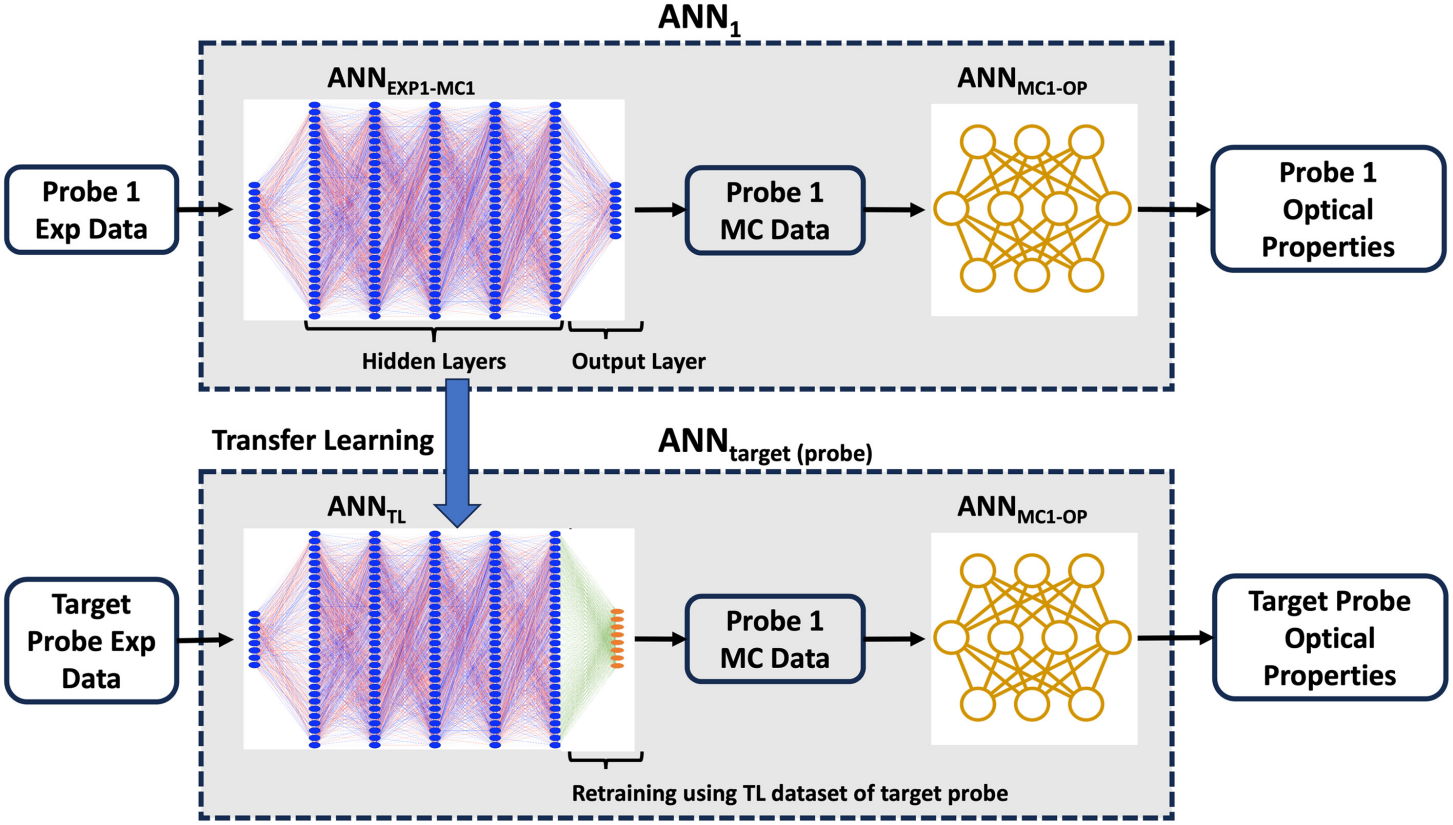
TL to create ${\mathrm{ANN}}_{\text{target}(\text{probe})}$ from ${\mathrm{ANN}}_{1}$ by retraining the output layer of ${\mathrm{ANN}}_{\mathrm{EXP}1\text{-}\mathrm{MC}1}$. Although the TL algorithm was applied to all ANN of the ${\mathrm{ANN}}_{\mathrm{EXP}1\text{-}\mathrm{MC}1}$ ensemble, only one ANN is shown here for clarity.

Here the ${\mathrm{ANN}}_{\mathrm{EXP}1\text{-}\mathrm{MC}1}$ network of ${\mathrm{ANN}}_{1}$ was used as the pretrained model. To accomplish probe-to-probe TL, only the output layer of the ${\mathrm{ANN}}_{\mathrm{EXP}1\text{-}\mathrm{MC}1}$ network was modified. The output layer of each individual ANN of the ${\mathrm{ANN}}_{\mathrm{EXP}1\text{-}\mathrm{MC}1}$ ensemble was retrained using a small TL dataset collected by the target probe to create an ${\mathrm{ANN}}_{\mathrm{TL}}$ ensemble for the target probe as shown in Fig. 3. To retrain each individual ANN, a train-validation split (80%:20%) was performed on the TL dataset with different random initializations. The validation dataset was used to prevent the resulting TL model from overfitting to the TL dataset using an early stopping callback (patience = 10).^16^ This low patience early stopping callback, coupled with a low learning rate ($10^{-4}$), prevented the TL model from experiencing “catastrophic forgetting,”^19^ which occurs when a network forgets previously learned information when adapting to new tasks, ensuring the preservation of knowledge learned from initial probe dataset. Although the maximum number of epochs was set to 500, the training process was terminated well before reaching this limit due to the early stopping callback.

The inputs for the TL dataset were experimental reflectance signals collected from selected TL phantoms with known optical properties described in Sec. 2.5.1, and the outputs were simulated reflectance for probe 1 obtained from the probe 1 MC library (${\mathrm{MC}}_{1}$) described in Sec. 2.5.2 for these known optical properties. Hence, the ${\mathrm{ANN}}_{\mathrm{TL}}$ ensemble essentially learned the mapping from the target probe’s experimental reflectance signal to the corresponding simulated reflectance signal for probe 1. This allowed us to reuse ${\mathrm{ANN}}_{\mathrm{MC}1\text{-}\mathrm{OP}}$, which eliminated the need for time-consuming generation of the MC library and subsequent ANN creation for the target probe. ${\mathrm{ANN}}_{\mathrm{TL}}$ was joined with ${\mathrm{ANN}}_{\mathrm{MC}1\text{-}\mathrm{OP}}$ described earlier to create an ${\mathrm{ANN}}_{\text{target}(\text{probe})}$ that took the target probe’s experimental reflectance intensities as inputs and outputs corresponding optical properties.

Successful TL relies on an effective TL strategy, which includes the size and diversity of the TL dataset, and optimal selection of hyperparameters (e.g., learning rate, epochs, and batch size). In order to assess our TL approach, we selected the performance of ${\mathrm{ANN}}_{1}$ on the test dataset ($E_{\mathrm{EXP}1\text{-}\mathrm{OP}1}$) as the benchmark. The effective validation of our TL model ${\mathrm{ANN}}_{\text{target}}$ was determined by its capability to attain performance levels on par with those of ${\mathrm{ANN}}_{1}$. Root-mean-squared error (RMSE) and mean-absolute percent error (MAPE) were used as the error metrics.

In our clinical context, it is impractical to collect a large number of calibration spectra, even though having a larger number of calibration spectra with diverse conditions in the TL dataset could be beneficial. Hence, to understand the effect of TL dataset size and its diversity on the TL performance, our TL technique was applied to the pretrained model (${\mathrm{ANN}}_{\mathrm{EXP}1\text{-}\mathrm{MC}1}$) with varying sized probe 2 TL datasets. Different subsets of the ${\mathrm{TL}}_{\mathrm{EXP}2\text{-}\mathrm{MC}1}$ dataset were used to create different TL models and their performance were evaluated on a test dataset ($E_{\mathrm{EXP}2\text{-}\mathrm{MC}1}$). This enabled us to determine which spectra should be incorporated into the TL dataset to ensure its effectiveness. In total, the ${\mathrm{TL}}_{\mathrm{EXP}2\text{-}\mathrm{MC}1}$ dataset consisted of 30 spectra. At first, we used the full ${\mathrm{TL}}_{\mathrm{EXP}2\text{-}\mathrm{MC}1}$ dataset to create the TL model. Subsequently, we reduced the size of the ${\mathrm{TL}}_{\mathrm{EXP}2\text{-}\mathrm{MC}1}$ dataset so that it only included phantoms with the lowest ($\mu_{a}=0.05\;{\mathrm{cm}}^{-1}$) and highest absorption ($\mu_{a}=1\;{\mathrm{cm}}^{-1}$) coefficient at 665 nm for each scattering condition, which resulted in 10 spectra. These spectra held particular importance for effective TL, as they spanned the outermost boundaries of the dataset. Subsequently, we used different smaller combinations of these 10 spectra for TL, down to a single spectrum.

The knowledge learned from this step allowed us to create a TL algorithm that included the required number and conditions of TL spectra, and optimal hyperparameter settings for successful probe-to-probe TL. To validate the generalizability and robustness of this TL algorithm, the exact algorithm was applied to a new probe (probe 3). The TL model was created using the ${\mathrm{TL}}_{\mathrm{EXP}3\text{-}\mathrm{MC}1}$ dataset and performance of the resulting TL model was evaluated on an independent test dataset ($E_{\mathrm{EXP}3\text{-}\mathrm{OP}3}$).

### 2.5 Datasets

#### 2.5.1 Phantom experiments and associated datasets

Three stages of phantom data were collected using three different probes. In all phantoms, methylene blue (Akorn, Inc., Lake Forest, Illinois, United States) was used as the absorber and Intralipid 20% (Fresenius Kabi AG, Bad Homburg, Germany) was used as the scatterer. Appropriate amounts of MB and Intralipid were mixed with 300 mL of distilled water (DI) in a black spray-painted container (Rust-Oleum Flat Black, Vernon Hills, Illinois, United States) to create the phantoms described in the sections “Probe 1 dataset,” “Probe 2 dataset,” and “Probe 3 dataset.” Concentration ranges of each component were selected to encompass the optical property range pertinent to our clinical application.^4^^,^^5^ Separate MB and Intralipid stocks were used for data collection for each separate probe, with each stock characterized as described below.

In order to calculate the necessary volumes of Intralipid and MB to add to DI water to achieve the desired optical properties, absorption ($\mu_{a}$) spectra of MB, scattering ($\mu_{s}$) spectra of Intralipid, and scattering anisotropy coefficients (g) for Intralipid were required. The first two were measured using a commercial spectrophotometer (Cary 50 Varian, Santa Clara, California, United States). To determine the scattering anisotropy of each Intralipid stock, we created 30 phantoms with $\mu_{s}=25$, 37.5, 62.5, 75, and $125\;{\mathrm{cm}}^{-1}$ at 665 nm, each of which had $\mu_{a}=0.05$, 0.1, 0.25, 0.5, 0.75, and $1.0\;{\mathrm{cm}}^{-1}$ at 665 nm from each stock. These phantoms were distinct from those used for training and/or validation. An iterative fitting algorithm was used to determine g by minimizing the difference between experimental signal and corresponding simulated signal obtained from the MC library described in Sec. 2.5.2. The calculated g values were similar to the literature values^20^^,^^21^ and showed small interstock variation. This small variation was relevant as it considerably affected the quality of fitting of the algorithm. This was expected because of the relatively small SDS present in our probes, making them considerably more sensitive to variations in scattering anisotropy. Hence, it was important for us to determine the value of g for each Intralipid stock rather than relying on the fixed values from the literature.

Our experimental phantom data collection process is briefly described here, the details of which can be found in our earlier work.^4^ We first allowed the source lamp to reach a steady state over ∼30  min. Calibration measurements were taken using a 3-in. calibration sphere to account for fiber throughput and lamp power variation. The probe was then placed in contact with the liquid phantom while continuously stirring. Light was emitted into the phantom using the source fiber, and diffuse reflectance spectra were collected through each of the detector fibers. For both the calibration measurements and phantom measurements, corresponding dark measurements were taken and subtracted. Then the dark corrected spectra were normalized by the corresponding dark corrected calibration data for each detection fiber to obtain the final DRS. Each spectrum consisted of 316 wavelengths (500 to 740 nm).

**Fig. 4:**
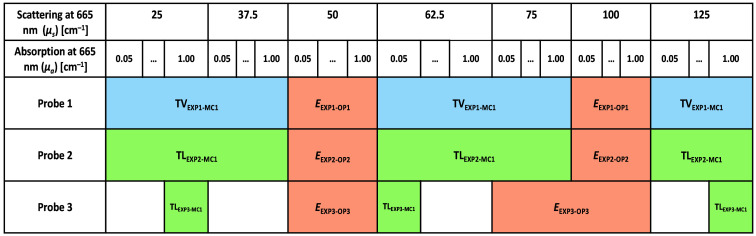
Data for different probes and their corresponding optical property ranges. The blue, green, and orange colors correspond to training, TL, and test datasets, respectively.

Sections “Probe 1 dataset,” “Probe 2 dataset,” and “Probe 3 dataset” detail phantom datasets and their optical properties where TV, TL, and E denote training and validation, TL, and testing datasets, respectively. Figure 4 visually represents these datasets with blue, green, and orange denoting training, TL, and test sets, respectively. The subscript associated with each dataset is divided into two parts, with the first part indicating the input and the second part indicating the output of each dataset. EXP, MC, and OP, respectively, denote experimental reflectance intensity, simulated reflectance intensity, and optical properties. The numerical value in the subscript specifies the probe or MC library utilized in generating the dataset.

##### Probe 1 dataset

Phantom data collected using probe 1 were used to create the two following datasets (see Fig. 4).

• ${\mathrm{TV}}_{\mathrm{EXP}1\text{-}\mathrm{MC}1}$ consisted of phantoms with $\mu_{s}=25$, 37.5, 62.5, 75, and $125\;{\mathrm{cm}}^{-1}$ at 665 nm, each of which had $\mu_{a}=0.05$, 0.1, 0.25, 0.5, 0.75, and $1.0\;{\mathrm{cm}}^{-1}$ at 665 nm (30 phantoms).
• $E_{\mathrm{EXP}1\text{-}\mathrm{OP}1}$ included phantoms with $\mu_{s}=50$ and $100\;{\mathrm{cm}}^{-1}$ at 665 nm, each of which had $\mu_{a}=0.05$, 0.1, 0.25, 0.5, 0.75, and $1.0\;{\mathrm{cm}}^{-1}$ at 665 nm (12 phantoms).

##### Probe 2 dataset

Phantom data collected using probe 2 were used to create the two following datasets (see Fig. 4).

• ${\mathrm{TL}}_{\mathrm{EXP}2\text{-}\mathrm{MC}1}$ consisted of phantoms with $\mu_{s}=25$, 37.5, 62.5, 75, and $125\;{\mathrm{cm}}^{-1}$ at 665 nm, each of which had $\mu_{a}=0.05$, 0.1, 0.25, 0.5, 0.75, and $1.0\;{\mathrm{cm}}^{-1}$ at 665 nm (30 phantoms).
• $E_{\mathrm{EXP}2\text{-}\mathrm{MC}1}$ and $E_{\mathrm{EXP}2\text{-}\mathrm{OP}2}$ included phantoms with $\mu_{s}=50$ and $100\;{\mathrm{cm}}^{-1}$ at 665 nm, each of which had $\mu_{a}=0.05$, 0.1, 0.25, 0.5, 0.75, and $1.0\;{\mathrm{cm}}^{-1}$ at 665 nm (12 phantoms).

##### Probe 3 dataset

Phantom data were collected using probe 3 to create the following two datasets (see Fig. 4).

• ${\mathrm{TL}}_{\mathrm{EXP}3\text{-}\mathrm{MC}1}$ comprised of the phantoms, which had $\mu_{s}=25\;{\mathrm{cm}}^{-1}$ and $\mu_{a}=1\;{\mathrm{cm}}^{-1}$, $\mu_{s}=62.5\;{\mathrm{cm}}^{-1}$ and $\mu_{a}=0.05\;{\mathrm{cm}}^{-1}$, and $\mu_{s}=125\;{\mathrm{cm}}^{-1}$ and $\mu_{a}=1\;{\mathrm{cm}}^{-1}$. All coefficients are given for 665 nm (3 phantoms).
• $E_{\mathrm{EXP}3\text{-}\mathrm{OP}3}$ consisted of phantoms with $\mu_{s}=50$, 75, and $100\;{\mathrm{cm}}^{-1}$ at 665 nm, each of which had $\mu_{a}=0.05$, 0.1, 0.25, 0.5, 0.75, and $1.0\;{\mathrm{cm}}^{-1}$ at 665 nm (18 phantoms).

#### 2.5.2 Monte Carlo dataset

A Monte Carlo (MC) model for probe 1 was constructed for optical property retrieval from diffuse reflectance spectra. A detailed account is of this is available in our previous publication.^4^ In summary, we generated the MC library (${\mathrm{MC}}_{1}$) by conducting GPU accelerated MC simulations across a wide range of parameter combinations, encompassing values for absorption coefficient ($\mu_{a}$) from 0.0001 to $25\;{\mathrm{cm}}^{-1}$ and scattering coefficient ($\mu_{s}$) from 1 to $250\;{\mathrm{cm}}^{-1}$. These simulations were carried out using a refractive index of n=1.37 and employed the Henyey–Greenstein phase function with a scattering anisotropy factor (g) of 0.7, resulting in reduced scattering coefficients ($\mu_{s}^{\prime }$) from 0.3 to $75\;{\mathrm{cm}}^{-1}$ for the MC library. The simulations were executed using $10^{8}\text{ photon}$ packets. The computations were performed on a Quadro RTX6000 GPU equipped with 24 GB of GPU memory (NVIDIA Corporation, Santa Clara, California, United States), which took about 7 days to run all optical property combinations. This MC library was used to create a ${\mathrm{TV}}_{\mathrm{MC}1\text{-}\mathrm{OP}1}$ dataset where the inputs were simulated reflectance intensity at eight fibers and outputs were corresponding absorption coefficients ($\mu_{a}$) and reduced scattering coefficients ($\mu_{s}^{\prime }$).

### 2.6 Statistical Analysis

Measured values are summarized as mean ± standard deviation. Paired comparisons were performed using the Wilcoxon matched-pairs signed rank test. Comparisons between the number of spectra in the TL dataset used the Kruskal–Wallis test, with Dunn’s test for multiple comparisons. GraphPad Prism (v10, GraphPad Software, Inc., Boston, Massachusetts, United States) was used for all statistical analysis.

## 3 Results

### 3.1 Performance of Initial Neural Network (${\mathrm{ANN}}_{1}$)

#### 3.1.1 Training and performance of ${\mathrm{ANN}}_{\mathrm{EXP}1\text{-}\mathrm{MC}1}$ ensemble

**Fig. 5:**
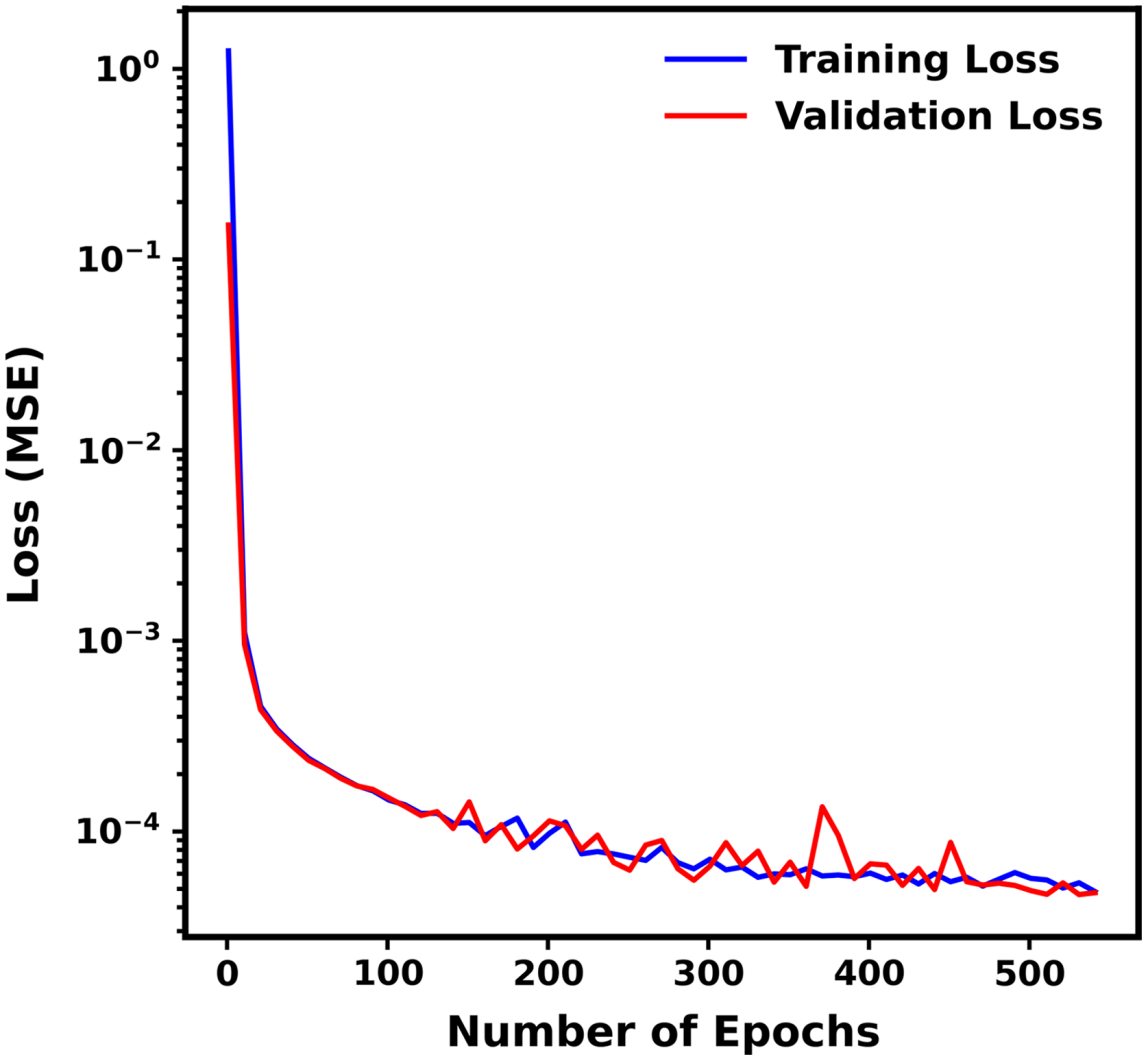
Loss (MSE) versus epochs for ANN #1 of the ${\mathrm{ANN}}_{\mathrm{EXP}1\text{-}\mathrm{MC}1}$ ensemble. Blue and red colors correspond to training and validation loss, respectively.

Figure 5 demonstrates the progression of both training and validation losses (MSE) against the number of epochs for a representative ANN (ANN #1) selected from the ${\mathrm{ANN}}_{\mathrm{EXP}1\text{-}\mathrm{MC}1}$ ensemble. The plot illustrates the convergence of the ANN as training proceeded, with training and validation losses closely aligning. Early stopping callback halted the training at 544 epochs.

**Fig. 6:**
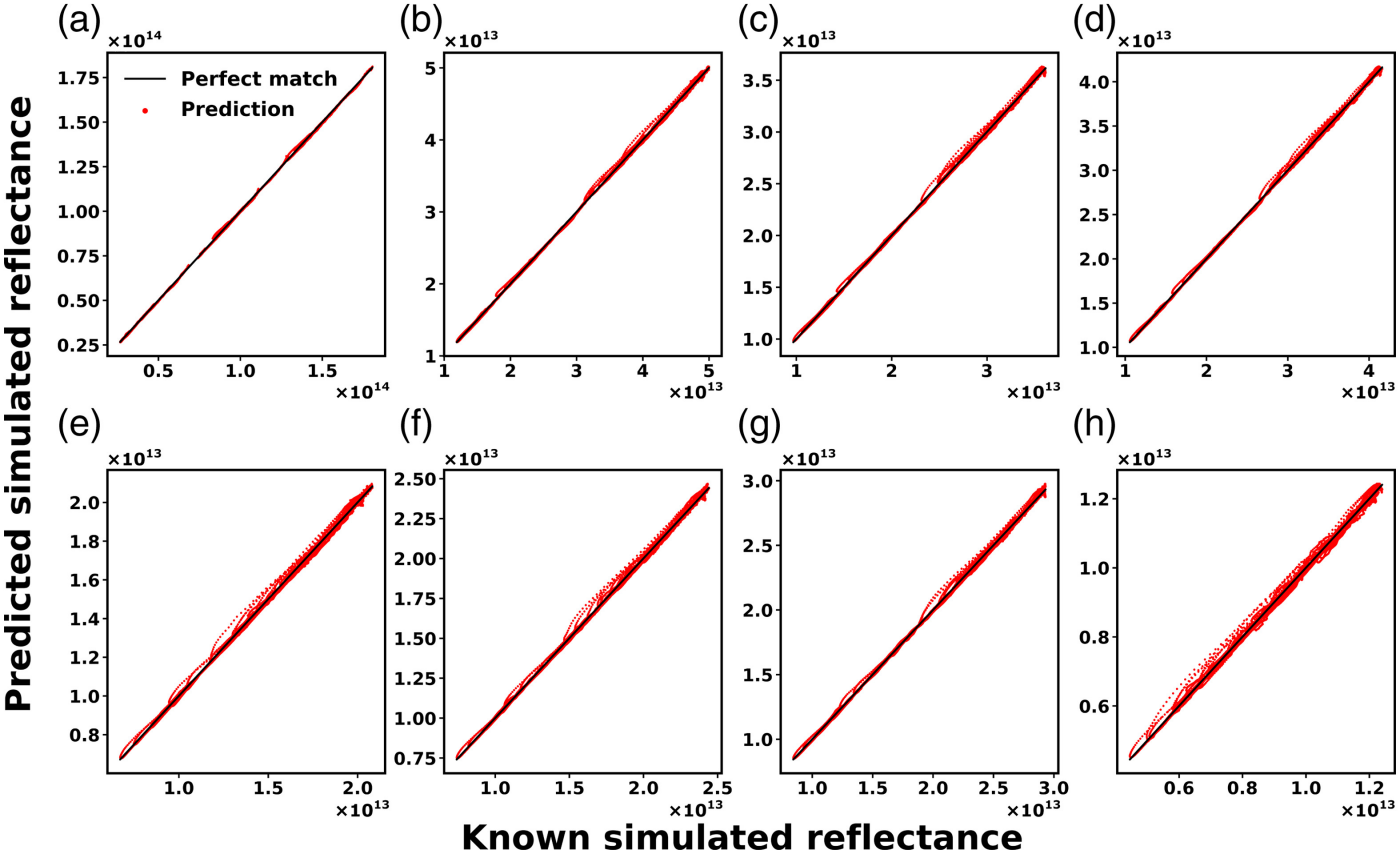
Performance of ${\mathrm{ANN}}_{\mathrm{EXP}1\text{-}\mathrm{MC}1}$ for (a)–(h) detector fibers 1 to 8. Red dots indicate predictions and solid black lines denote perfect prediction. The units for simulated reflectance signals are detected photon weight.

The performance of the ${\mathrm{ANN}}_{\mathrm{EXP}1\text{-}\mathrm{MC}1}$ ensemble was evaluated on the entire ${\mathrm{TV}}_{\mathrm{EXP}1\text{-}\mathrm{MC}1}$ dataset to ensure that it properly captured the relationship between experimental and simulated reflectance signals across all detector fibers. Figure 6 demonstrates that the ensemble accurately learned the relationship between input and outputs. The RMSE and MAPE values were small across all fibers, as detailed in Table 2. The ensemble’s performance was evaluated on the entire ${\mathrm{TV}}_{\mathrm{EXP}1\text{-}\mathrm{MC}1}$ dataset.

**Table 2:** RMSE and MAPE for predicted simulated reflectance signal across eight detector fibers.

Metric	Detector fiber 1	Detector fiber 2	Detector fiber 3	Detector fiber 4	Detector fiber 5	Detector fiber 6	Detector fiber 7	Detector fiber 8
RMSE	$5.34\times 10^{11}$	$2.18\times 10^{11}$	$1.80\times 10^{11}$	$1.99\times 10^{11}$	$1.36\times 10^{11}$	$1.45\times 10^{11}$	$1.59\times 10^{11}$	$1.05\times 10^{11}$
MAPE (%)	0.59	0.54	0.55	0.55	0.62	0.59	0.57	0.85

#### 3.1.2 Training and performance of ${\mathrm{ANN}}_{\mathrm{MC}1\text{-}\mathrm{OP}}$ ensemble

**Fig. 7:**
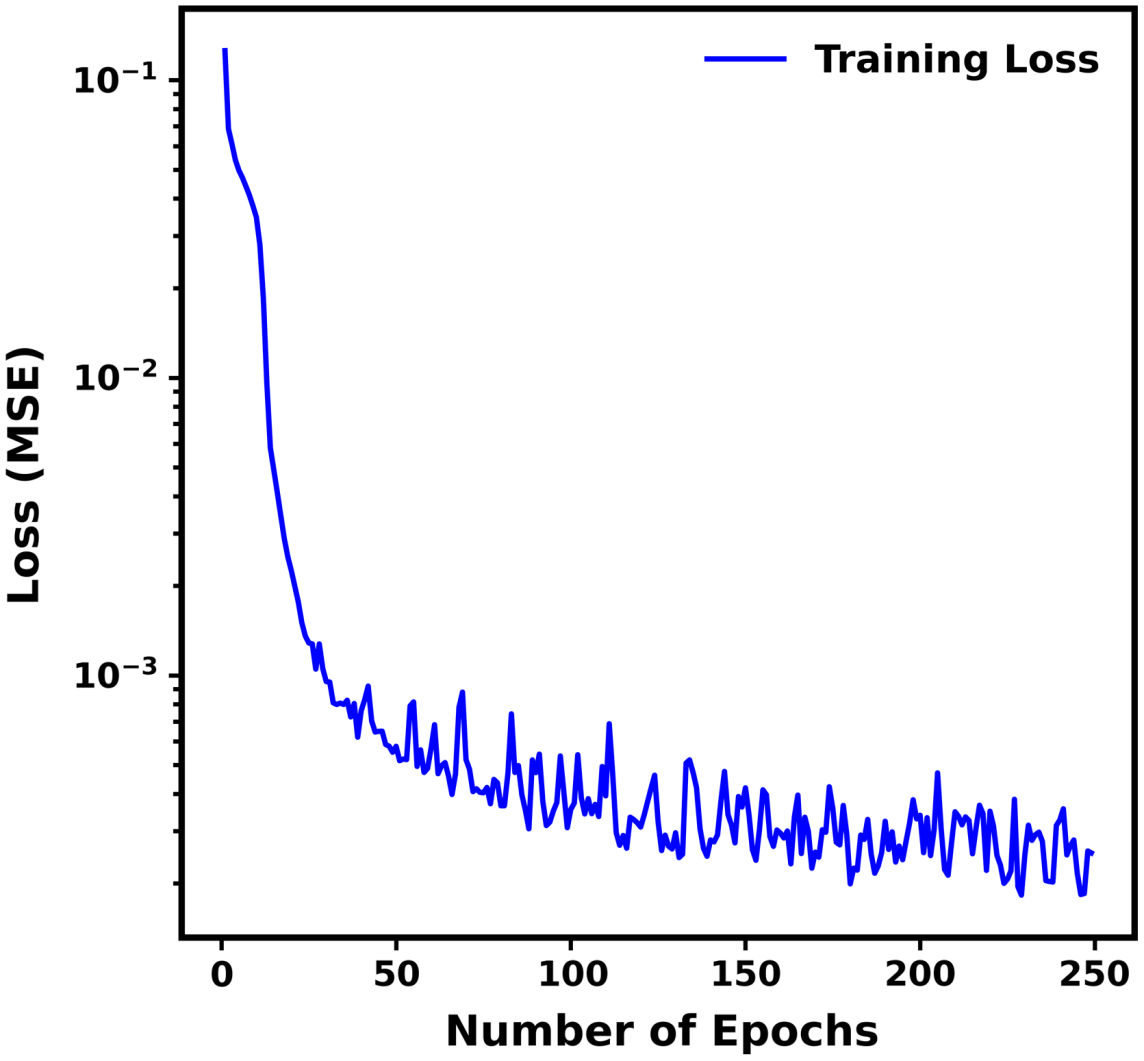
Loss (MSE) versus epoch for ${\mathrm{ANN}}_{\mathrm{MC}1\text{-}\mathrm{OP}}$ ensemble. Blue color corresponds to training loss.

Figure 7 shows the training loss (MSE) versus number of epochs for the ${\mathrm{ANN}}_{\mathrm{MC}1\text{-}\mathrm{OP}}$ ensemble, demonstrating training convergence.

**Fig. 8:**
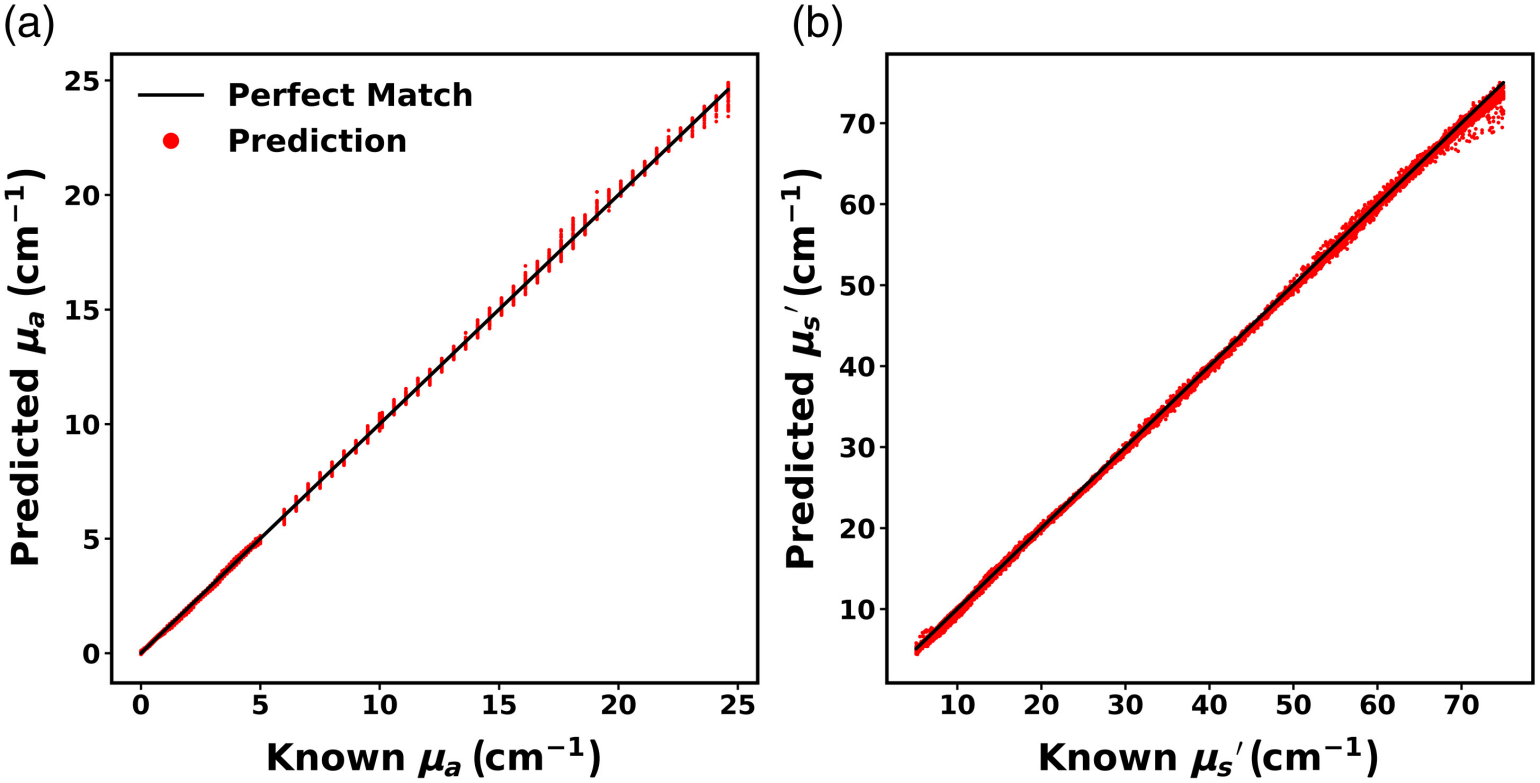
Predicted (a) absorption coefficients ($\mu_{a}$) and (b) reduced scattering coefficients ($\mu_{s}^{\prime }$). Red dots represent predictions and solid black lines denote perfect prediction.

The performance of the ${\mathrm{ANN}}_{\mathrm{MC}1\text{-}\mathrm{OP}}$ ensemble on its test set is depicted in Fig. 8. Absorption coefficient ($\mu_{a}$) was predicted with a small $\mathrm{RMSE}=0.087\;{\mathrm{cm}}^{-1}$ (MAPE=290%). This elevated MAPE can be attributed to discrepancies arising from very small absorption coefficients ($\mu_{a}<0.01\;{\mathrm{cm}}^{-1}$). For $\mu_{a}>0.01\;{\mathrm{cm}}^{-1}$, RMSE was $0.091\;{\mathrm{cm}}^{-1}$ (MAPE=6.4%). Reduced scattering coefficient ($\mu_{s}^{\prime }$) was predicted with a small $\mathrm{RMSE}=0.48\;{\mathrm{cm}}^{-1}$ (MAPE=1.1%). These low RMSE values indicate that ${\mathrm{ANN}}_{\mathrm{MC}1\text{-}\mathrm{OP}}$ successfully learned the relationship between simulated reflectance intensity and corresponding optical properties.

#### 3.1.3 Performance of the initial ANN (${\mathrm{ANN}}_{1}$)

**Fig. 9:**
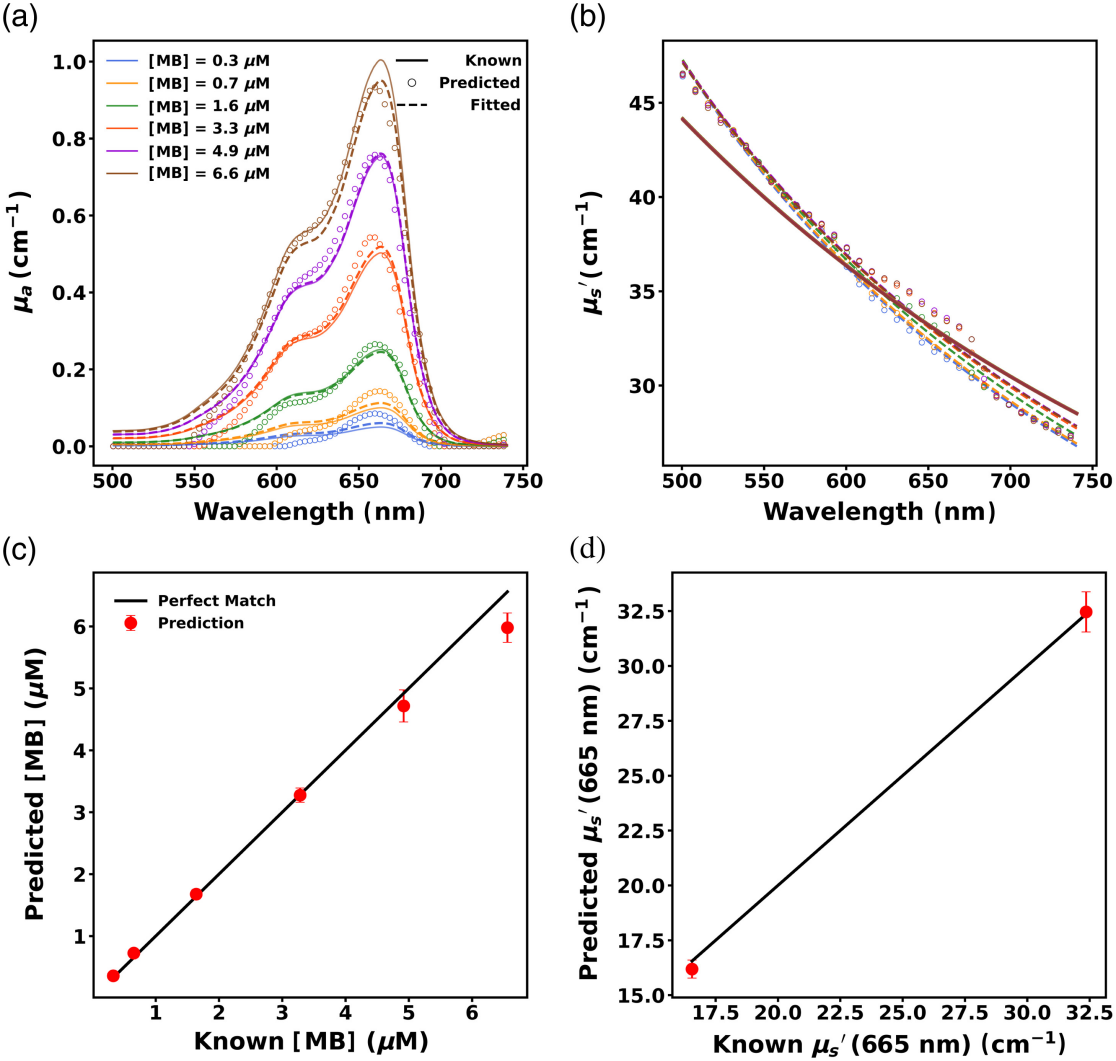
Predicted (a) absorption spectra and (b) reduced scattering spectra for a representative phantom. Solid lines, open circles, and dashed lines correspond to known, predicted, and fitted spectra, respectively, whereas colors indicate MB concentration. Predicted (c) MB concentration and (d) $\mu_{s}^{\prime }$ at 665 nm across phantoms. Predictions are denoted by red dots and solid black lines denote perfect prediction. Symbols indicate mean recovered values across all phantom measurements at that value, and error bars correspond to standard deviation across phantoms with identical optical properties. Error bars are included in all cases but are not always visible.

The performance of the initial ANN (${\mathrm{ANN}}_{1}$) on the test set ($E_{\mathrm{EXP}1\text{-}\mathrm{OP}1}$) is presented in Fig. 9. The absorption and reduced scattering spectra predictions from ${\mathrm{ANN}}_{1}$ for a representative phantom (with $\mu_{s}=100\;{\mathrm{cm}}^{-1}$ at 665 nm and $\mu_{a}$ values of 0.05, 0.1, 0.25, 0.5, 0.75, and $1.0\;{\mathrm{cm}}^{-1}$ at 665 nm) from the test dataset are illustrated in Figs. 9(a) and 9(b). The predicted absorption and reduced scattering spectra were fitted using the procedure mentioned in Sec. 2.3.2, resulting in highly accurate fitting.

Figure 9(c) illustrates that ${\mathrm{ANN}}_{1}$ accurately predicted the MB concentration of the phantoms in the test dataset with a small RMSE=0.29  μM (MAPE = 7.46%). It also performed well in predicting the reduced scattering coefficient ($\mu_{s}^{\prime }$) across all wavelengths, as evidenced by a low $\mathrm{RMSE}=1.08\;{\mathrm{cm}}^{-1}$ (MAPE=3.6%). The reduced scattering coefficient at 665 nm ($\mu_{s,665}^{\prime }$) was predicted with $\mathrm{RMSE}=0.77\;{\mathrm{cm}}^{-1}$ (MAPE=2.54%), which is shown in Fig. 9(d).

### 3.2 Transfer Learning

#### 3.2.1 Performance of initial ANN (${\mathrm{ANN}}_{1}$) on Probe 2 data without transfer learning

**Fig. 10:**
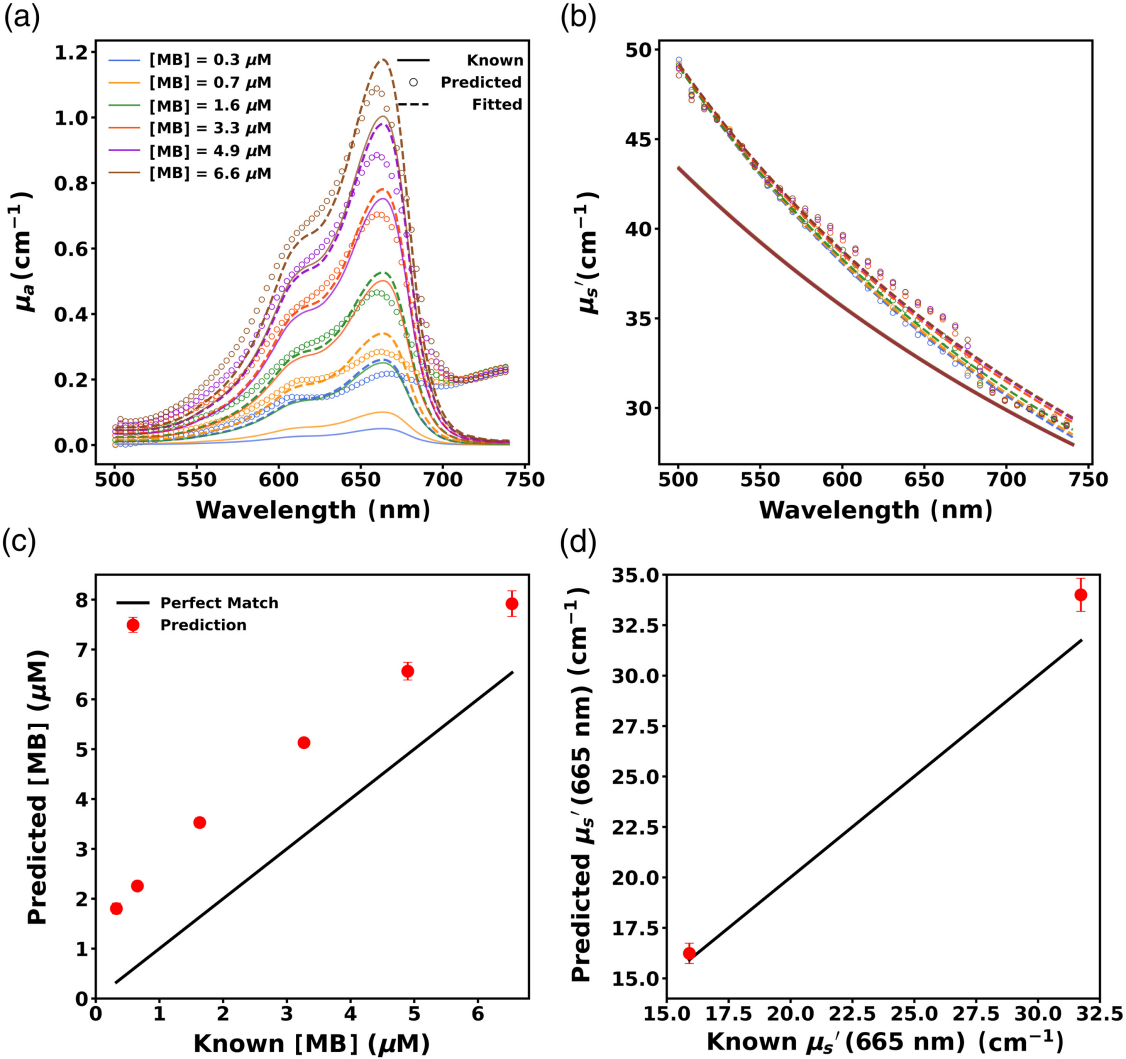
Predicted (a) absorption spectra and (b) reduced scattering spectra for a representative phantom measured with probe 2 using the neural network trained on probe 1 data (${\mathrm{ANN}}_{1}$) show poor quality extraction of optical properties. Solid lines, open circles, and dashed lines correspond to known, predicted, and fitted spectra, respectively, and colors indicate MB concentration. Predicted (c) MB concentration and (d)  $\mu_{s}^{\prime }$ at 665 nm across phantoms also show poor performance, where predictions are denoted by red dots and solid black lines denote perfect prediction. Error bars are included in all cases but are not always visible.

To assess the performance of ${\mathrm{ANN}}_{1}$ for data collected with another probe, we evaluated its performance on the $E_{\mathrm{EXP}2\text{-}\mathrm{OP}2}$ dataset. Figures 10(a) and 10(b) illustrate the absorption and reduced scattering spectra predictions of ${\mathrm{ANN}}_{1}$ for a representative phantom from $E_{\mathrm{EXP}2\text{-}\mathrm{OP}2}$. ${\mathrm{ANN}}_{1}$ predicted much higher absorption than known absorption for this phantom as evident from Fig. 10(a). The MB concentration and fitted reduced scattering spectra were obtained by the same methods described in Sec. 3.1.3.

The failure of ${\mathrm{ANN}}_{1}$ to accurately predict MB concentration from probe 2 data can be seen in Fig. 10(c), which shows the performance of ${\mathrm{ANN}}_{1}$ for the entire $E_{\mathrm{EXP}2\text{-}\mathrm{OP}2}$ dataset. The MB concentration of phantoms was recovered with a large RMSE=1.67  μM (MAPE = 154.84%). It also performed worse in predicting the reduced scattering coefficient ($\mu_{s}^{\prime }$) across all wavelengths as evidenced by comparably higher $\mathrm{RMSE}=2.2\;{\mathrm{cm}}^{-1}$ (MAPE=4.85%). The reduced scattering coefficient at 665 nm ($\mu_{s,665}^{\prime }$) was predicted with $\mathrm{RMSE}=1.76\;{\mathrm{cm}}^{-1}$ (MAPE=5.14%), as shown in Fig. 10(d). These results indicate that employing ${\mathrm{ANN}}_{1}$, which was trained using probe 1 data, for optical property extraction from data collected by probe 2 results in large errors in predictions, particularly for absorption.

#### 3.2.2 Feature extraction

From the results of Sec. 3.2.1, it is evident that usage of the initial ANN (${\mathrm{ANN}}_{1}$) for probe 2 data leads to highly inaccurate prediction of optical properties. To adapt ${\mathrm{ANN}}_{1}$ to probe 2, we employed a feature extraction TL technique.

##### Number of training spectra versus performance

**Fig. 11:**
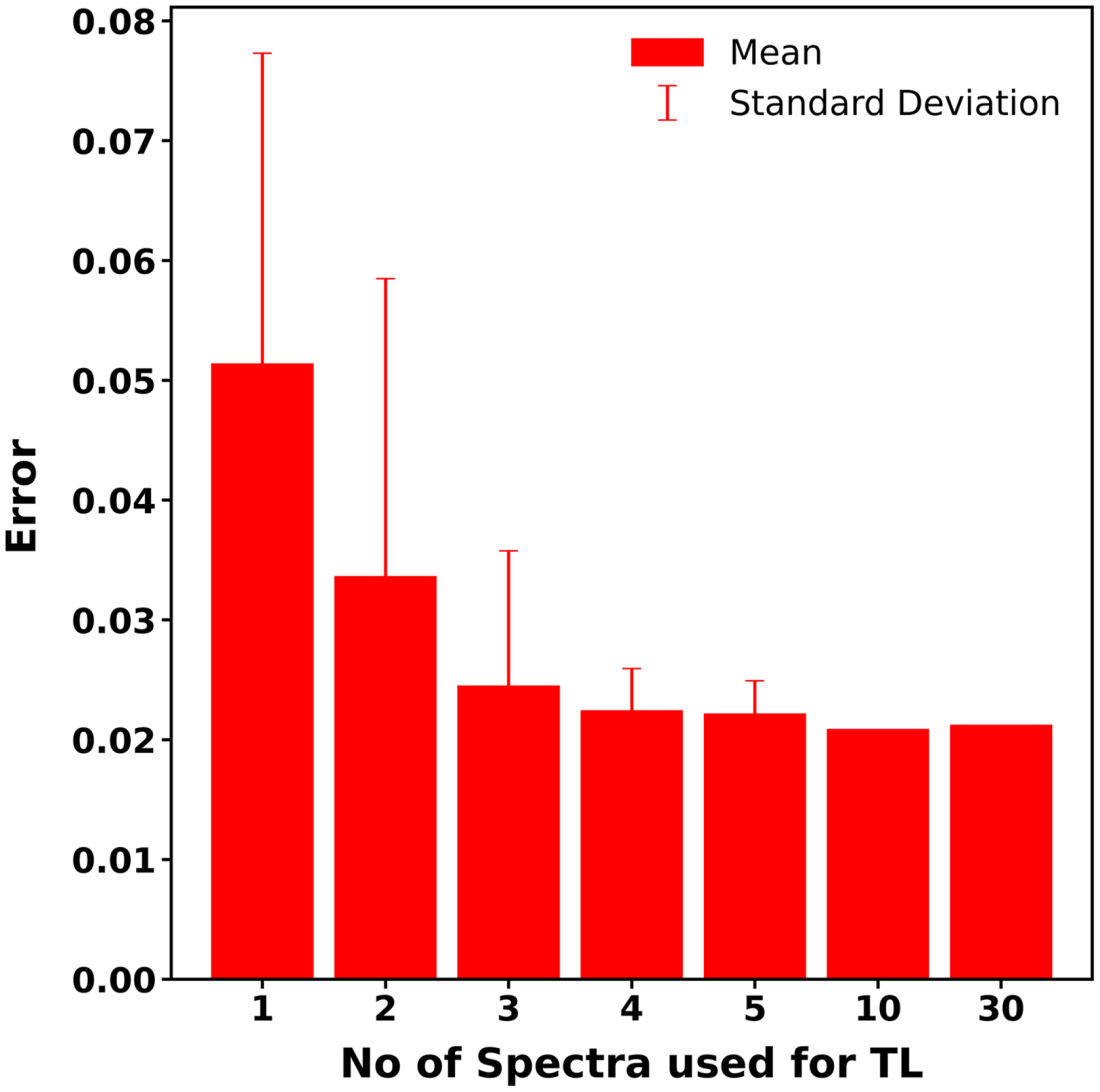
Prediction error for ${\mathrm{ANN}}_{\mathrm{TL}}$ models created from TL datasets with varying number of training spectra. The X axis denotes the number of spectra in the TL dataset, and the Y axis denotes the prediction error. Solid bars represent mean values across all combinations of the specified number of spectra, with error bars corresponding to standard deviation.

Figure 11 presents the prediction error for TL models created from different-sized TL training datasets on the $E_{\mathrm{EXP}2\text{-}\mathrm{MC}1}$ dataset. Initially, we employed 30 spectra, constituting the entire ${\mathrm{TL}}_{\mathrm{EXP}2\text{-}\mathrm{MC}1}$ dataset, to establish our baseline performance (error = 0.021). Subsequently, we reduced the TL dataset to 10 spectra, consisting of the lowest ($\mu_{a}=0.05\;{\mathrm{cm}}^{-1}$) and highest absorption ($\mu_{a}=1\;{\mathrm{cm}}^{-1}$) coefficients at 665 nm for each scattering condition. These 10 spectra were selected as they constitute the outermost boundaries of the ${\mathrm{TL}}_{\mathrm{EXP}2\text{-}\mathrm{MC}1}$ dataset. Figure 11 demonstrates that using these 10 spectra for TL leads to identical error compared to that of using all 30 spectra (0.021 versus 0.021), indicating that the 20 intermediate spectra can be discarded from the TL dataset without loss in the performance.

Smaller subsets of these 10 spectra were then used for TL, ranging from 1 to 5 spectra. Prediction error for a given number of spectra was calculated for all combinations selected from these 10 spectra, in order to determine mean error. Figure 11 illustrates that performance worsens as we employ fewer spectra for TL. Notably, there is a significant difference in error between using 2 and 3 spectra (0.034 versus 0.024, p=0.0009), whereas the error between 3 and 4 spectra was not significantly different (0.024 versus 0.022, p>0.99). We therefore included three spectra in our TL dataset.

##### Selection of spectra and creation of TL algorithm

To select the optimal three spectra to be incorporated into the TL dataset, we considered the strengths and weaknesses of ANNs. ANNs work accurately and reliably only within the range of the data they were trained on. Beyond this range, performance can degrade quite rapidly.^22^ Hence, to maximize the range of validity of our TL model, we decided to integrate two spectra from the extremes of the TL dataset (${\mathrm{TL}}_{\mathrm{EXP}2\text{-}\mathrm{MC}1}$). Specifically, we included the spectrum with the lowest scattering and highest absorption ($\mu_{s}=25\;{\mathrm{cm}}^{-1}$ and $\mu_{a}=1\;{\mathrm{cm}}^{-1}$ at 665 nm), as well as the spectrum with the highest scattering and absorption ($\mu_{s}=125\;{\mathrm{cm}}^{-1}$ and $\mu_{a}=1\;{\mathrm{cm}}^{-1}$ at 665 nm). Moreover, we also included an intermediate spectrum ($\mu_{s}=62.5\;{\mathrm{cm}}^{-1}$ and $\mu_{a}=0.05\;{\mathrm{cm}}^{-1}$ at 665 nm) to improve interpolation. In this step, we also determined the optimal hyperparameters for TL (e.g., learning rate and epochs).

Based on these, a TL algorithm was created for probe-to-probe TL, which will allow us to create an ${\mathrm{ANN}}_{\text{target}(\text{probe})}$ for the target probe optical property recovery.

The algorithm was

(1) Collection of three spectra from three known phantoms using the target probe to create the TL dataset.

The phantoms are

(a) $\mu_{s}=25\;{\mathrm{cm}}^{-1}$ and $\mu_{a}=1\;{\mathrm{cm}}^{-1}$ at 665 nm;
(b) $\mu_{s}=62.5\;{\mathrm{cm}}^{-1}$ and $\mu_{a}=0.05\;{\mathrm{cm}}^{-1}$ at 665 nm;
(c) $\mu_{s}=125\;{\mathrm{cm}}^{-1}$ and $\mu_{a}=1\;{\mathrm{cm}}^{-1}$ at 665 nm.

(2) Usage of the TL dataset to adapt ${\mathrm{ANN}}_{\mathrm{EXP}1\text{-}\mathrm{MC}1}$ to create ${\mathrm{ANN}}_{\mathrm{TL}}$ adapted for target probe.
(3) Integration of ${\mathrm{ANN}}_{\mathrm{TL}}$ with ${\mathrm{ANN}}_{\mathrm{MC}1\text{-}\mathrm{OP}}$ to create ${\mathrm{ANN}}_{\text{target}(\text{probe})}$, which is capable of recovering optical properties from DRS collected by the target probe.

##### Performance of ${\mathrm{ANN}}_{\text{target}(2)}$ on Probe 2 data

**Fig. 12:**
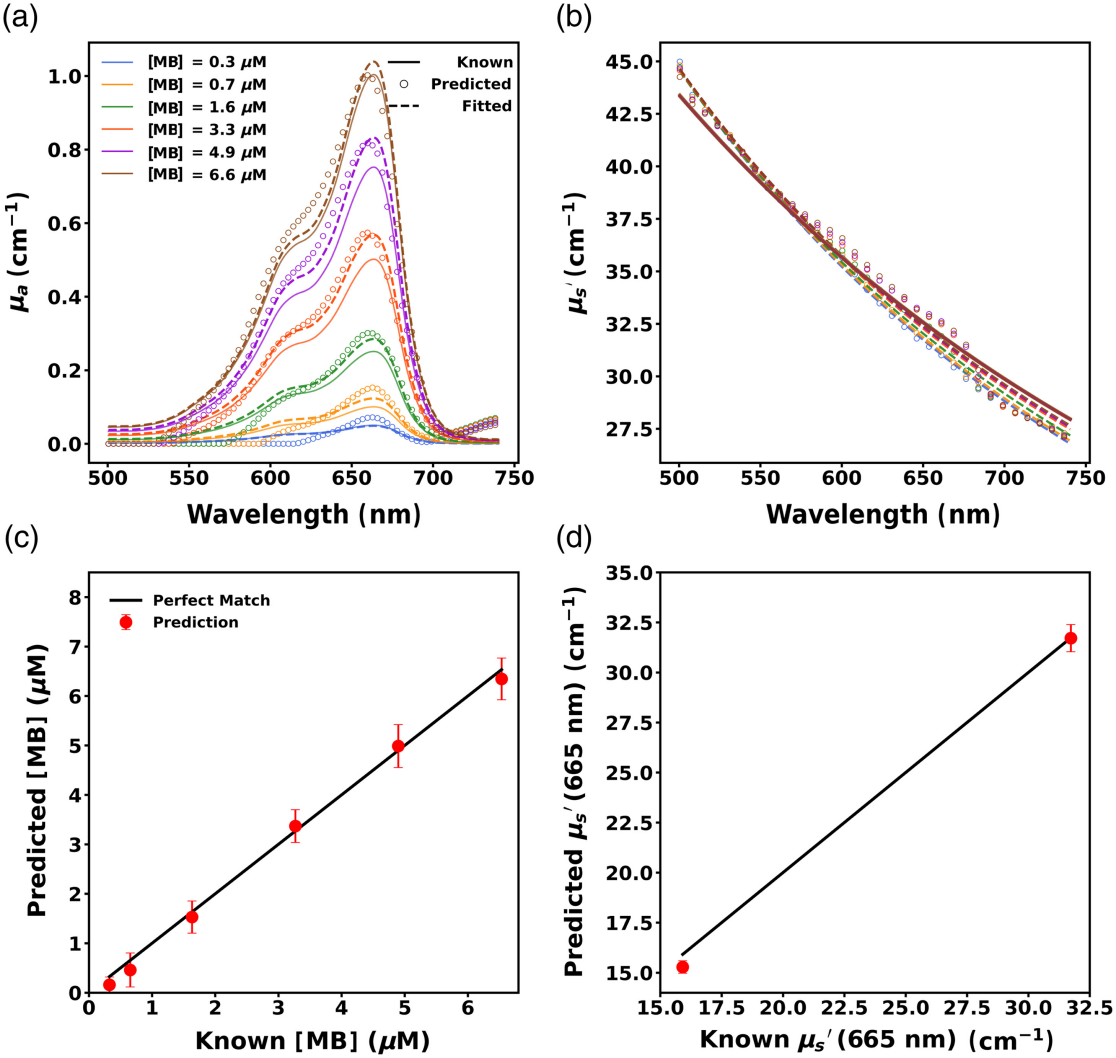
Predicted (a) absorption spectra and (b) reduced scattering spectra for a representative phantom measured with probe 2 after TL. Solid lines, open circles, and dashed lines correspond to known, predicted, and fitted spectra, respectively, whereas colors indicate MB concentration. Predicted (c) MB concentration and (d)  $\mu_{s}^{\prime }$ at 665 nm across phantoms, where predictions are denoted by red dots and solid black lines denote perfect prediction. Error bars are included in all cases but are not always visible.

This algorithm was applied to ${\mathrm{ANN}}_{1}$ to create ${\mathrm{ANN}}_{\text{target}(2)}$ for probe 2 and its performance was evaluated on $E_{\mathrm{EXP}2\text{-}\mathrm{OP}2}$. Figures 12(a) and 12(b) illustrate the absorption and reduced scattering spectra predictions of ${\mathrm{ANN}}_{\text{target}(2)}$ for a representative phantom from $E_{\mathrm{EXP}2\text{-}\mathrm{OP}2}$. We observe much better agreement between the known and predicted spectra for both absorption and reduced scattering compared to the incorrect insertion of probe 2 data into ${\mathrm{ANN}}_{1}$.

The ability of ${\mathrm{ANN}}_{\text{target}(2)}$ to accurately predict MB concentration can be seen in Fig. 12(c), which shows the performance of ${\mathrm{ANN}}_{\text{target}}$ for the entire $E_{\mathrm{EXP}2\text{-}\mathrm{OP}2}$ dataset. The MB concentration of phantoms was recovered with a small RMSE=0.38  μM (MAPE = 24.82%). It also showed superior performance in predicting the reduced scattering coefficient ($\mu_{s}^{\prime }$) across all wavelengths as evidenced by low $\mathrm{RMSE}=0.88\;{\mathrm{cm}}^{-1}$ (MAPE = 3.68%). The reduced scattering coefficient at 665 nm ($\mu_{s,665}^{\prime }$) was predicted with $\mathrm{RMSE}=0.71\;{\mathrm{cm}}^{-1}$ (MAPE = 3.01%), which is shown in Fig. 12(d).

Overall, ${\mathrm{ANN}}_{\text{target}(2)}$ exhibited significantly improved performance on the $E_{\mathrm{EXP}2\text{-}\mathrm{OP}2}$ dataset in comparison to ${\mathrm{ANN}}_{1}$ both in predicting MB concentration (p=0.0005) and reduced scattering coefficient at 665 nm (p=0.0005).

#### 3.2.3 Application of TL algorithm to Probe 3

**Fig. 13:**
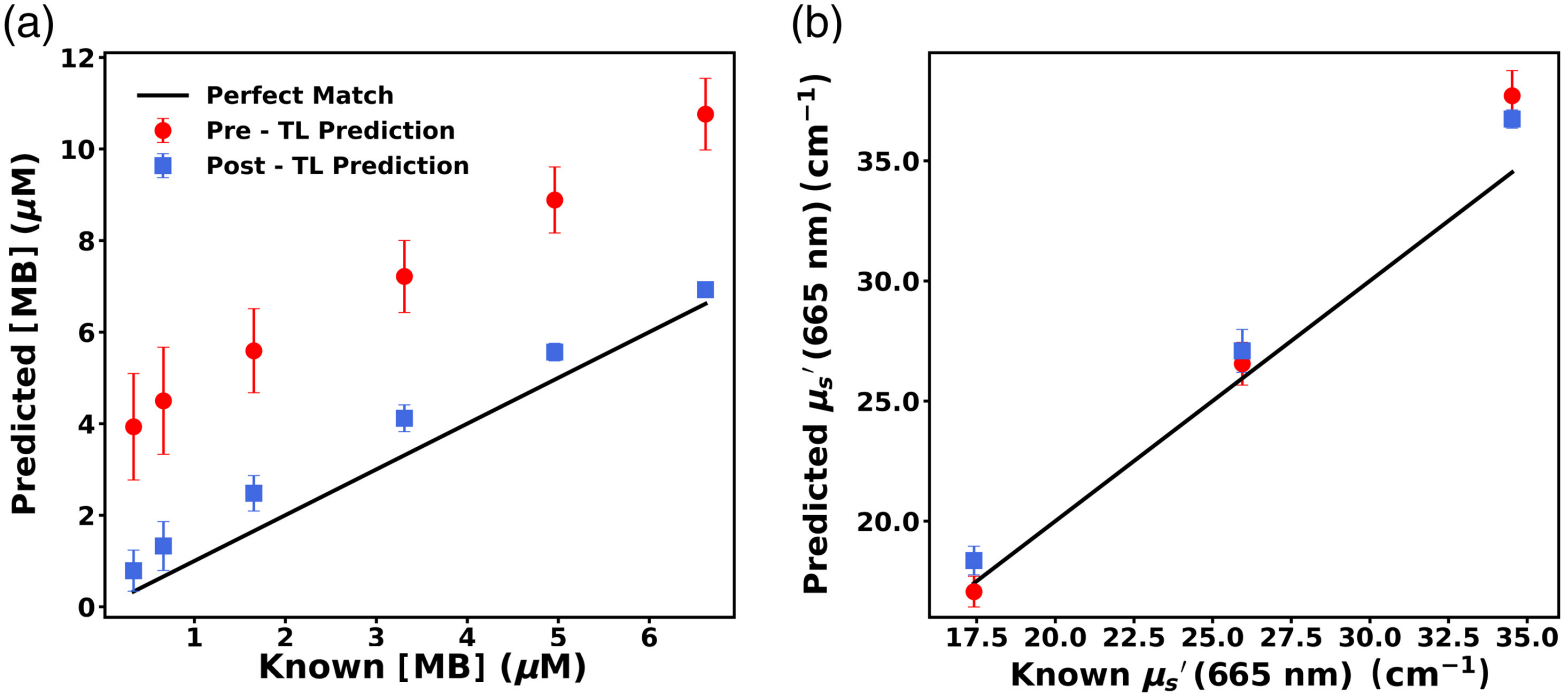
Predicted (a) MB concentration and (b)  $\mu_{s}^{\prime }$ at 665 nm for phantoms measured with probe 3 for both before (pre-TL) and after (post-TL) the application of TL. Pre-TL and post-TL predictions are denoted by red dots and blue squares, respectively, whereas solid black lines denote perfect prediction. Error bars are included in all cases but are not always visible.

The performance of ${\mathrm{ANN}}_{1}$ on probe 3 data ($E_{\mathrm{EXP}3\text{-}\mathrm{OP}3}$) is shown in Figs. 13(a) and 13(b) as the pre-TL prediction. The MB concentration prediction error was huge, with a RMSE=4.1  μM (MAPE=361.08%). For phantoms with $\mu_{a}>0.1\;{\mathrm{cm}}^{-1}$, MB concentration was predicted with RMSE=4.06  μM (MAPE=124.51%). Prediction of the reduced scattering coefficient ($\mu_{s}^{\prime }$) across all wavelengths was also poor, with $\mathrm{RMSE}=2.47\;{\mathrm{cm}}^{-1}$ (MAPE = 5.68%). The reduced scattering coefficient at 665 nm ($\mu_{s,665}^{\prime }$) was predicted with an $\mathrm{RMSE}=2.08\;{\mathrm{cm}}^{-1}$ (MAPE = 5.45%) as shown in Fig. 13(b).

The TL algorithm described in the section “Selection of spectra and creation of TL algorithm” was applied to ${\mathrm{ANN}}_{1}$ to create ${\mathrm{ANN}}_{\text{target}(3)}$ for probe 3 and its performance was also evaluated on $E_{\mathrm{EXP}3\text{-}\mathrm{OP}3}$, which are shown in Figs. 13(a) and 13(b) as the post-TL prediction. The MB concentration of phantoms was recovered with a RMSE=0.73  μM (MAPE=61.89%), as depicted in Fig. 13(a). This high MAPE can be attributed to mismatch in prediction of low-absorption phantoms for which slight mismatch in prediction causes huge MAPE values. For phantoms with $\mu_{a}>0.1\;{\mathrm{cm}}^{-1}$, MB concentration was predicted with RMSE=0.72  μM (MAPE=22.8%). It was also able to predict reduced scattering coefficient ($\mu_{s}^{\prime }$) across all wavelengths with a low $\mathrm{RMSE}=1.67\;{\mathrm{cm}}^{-1}$ (MAPE=4.11%). The reduced scattering coefficient at 665 nm ($\mu_{s,665}^{\prime }$) was predicted with $\mathrm{RMSE}=1.68\;{\mathrm{cm}}^{-1}$ (MAPE=5.43%), which is shown in Fig. 13(b).

${\mathrm{ANN}}_{\text{target}(3)}$ exhibited significantly improved performance on the $E_{\mathrm{EXP}3\text{-}\mathrm{OP}3}$ dataset in comparison to ${\mathrm{ANN}}_{1}$ for MB concentration prediction (p<0.0001), whereas prediction of the reduced scattering coefficient at 665 nm was not significantly improved (p=0.2).

## 4 Discussion

In this study, we created and validated an ANN that can accurately predict optical properties from DRS collected by a specific fiber-optic probe. Then we investigated utilization of this ANN for optical property prediction from DRS collected by other probes having dissimilar SDS. We found that doing so led to large errors in prediction. To solve this issue, we utilized a feature-extraction-based TL algorithm. We showed that this algorithm can reliably adapt an ANN created for one probe to a target probe for accurate optical property recovery by the target probe. This probe-to-probe calibration can be achieved using a small TL dataset consisting of only three spectra collected by the target probe.

Other groups have used a similar ANN approach for optical property recovery from DRS. Ivančič et al.^23^ predicted $\mu_{a}$ with $\mathrm{RMSE}=0.09\;{\mathrm{cm}}^{-1}$ (relative RMSE=2.6%) and $\mu_{s}^{\prime }$ with $\mathrm{RMSE}=0.26\;{\mathrm{cm}}^{-1}$ (relative RMSE=1.6%) at SDS ranging from 220 to 1200  μm. However, their performance evaluation was limited to a single low scattering and high absorption phantom. Furthermore, they employed separate ANNs for predicting each optical parameter, which resulted in four distinct ANNs, whereas we used a single ANN for predicting both absorption and reduced scattering spectra. Chong et al.^22^ demonstrated recovery of $\mu_{a}$ and $\mu_{s}^{\prime }$ with mean relative error ranging from 1.62% to 5.92% and 0.8% to 1.6%, respectively, with their baseline neural network and its physics-guided variants but only for a simulation dataset. An et al.^24^ developed ANNs for their spectroscopy system, which predicted $\mu_{a}$ with mean absolute error ranging from 0.13 to $0.17\;{\mathrm{cm}}^{-1}$ and $\mu_{s}^{\prime }$ ranging from 1.43 to $1.71\;{\mathrm{cm}}^{-1}$. However, their ANN prediction was limited to only four wavelengths compared to our broad-spectrum prediction ranging from 500 to 740 nm. Lan et al.^14^ predicted $\mu_{a}$ with Euclidean distance = 0.25 and $\mu_{s}^{\prime }$ with Euclidean distance = 4.14, which is somewhat difficult to interpret. With our initial ANN (${\mathrm{ANN}}_{1}$), we achieved comparable performance to these previous studies. However, these prior publications only investigated the performance of ANN-based optical property recovery for a single instance of their respective fiber-optic probes. The effects of SDS mismatch between probes on the recovered optical properties were not previously explored. This is vitally important for applications requiring multiple copies of a designed fiber-optic probe or single use devices.

We have shown that ANNs for optical property extraction are probe specific. In other words, an ANN created for one probe cannot be used for another probe if their SDS are considerably different. This is particularly true for the short SDS required by our clinical fiber optic probe. Precise knowledge of SDS is, therefore, required for traditional methods, such as MCLUTs^4^^,^^11^ or analytical approximations,^10^ as well as generation of Monte Carlo models for ANNs trained on simulation data. Hence, each time we intend to extract optical properties using a new probe, we are required to perform time-consuming probe characterization, probe-specific MC library creation, and phantom data collection, as well as ANN creation and validation to ensure satisfactory performance with this new probe. These steps require a substantial amount of effort, technical expertise, and time (7 to 14 days). These factors can severely limit the clinical implementation of an ANN-based spectroscopy system for our envisioned multicenter clinical trials or to transform this system into a widely used product on a large scale.^25^

As we have demonstrated, our TL algorithm offers a possible solution to these problems. It eliminates the need for performing the aforementioned lengthy and time-consuming probe-specific MC library and ANN creation for each new probe, reducing the time and expertise required. Even without code optimization, our probe-to-probe calibration can be done rapidly within 10 min, so it can be easily integrated into a clinical workflow. We envision that this would utilize well-characterized solid phantoms for TL, which has been shown to be highly reproducible.^26^ Furthermore, this TL algorithm could potentially reduce the cost of probe manufacturing. As we have shown that our algorithm allows for accurate recovery of data from probes with relatively large differences in SDS from those specified, this could allow for the utilization of less expensive probes with looser tolerances on fiber positioning. This could be extended to the utilization of sterile disposable probes for straightforward integration into the clinical workflow.

We observed significantly better prediction performance after the application of the TL algorithm for both probes 2 and 3. For probe 2, errors in both MB concentration and scattering coefficient prediction were reduced up to 77.2% and 60%, respectively. For probe 3, errors in both MB concentration and scattering coefficient prediction were reduced up to 82.2% and 32.4%, respectively. These findings indicate that the TL algorithm enhanced the prediction accuracy of both probes 2 and 3, demonstrating the generalizability of our proposed TL algorithm.

We have shown that the magnitude of prediction error depends on the SDS mismatch between the initial probe and the target probe. This was apparent when data from probe 2 or 3 were used as inputs to the initial ANN (${\mathrm{ANN}}_{1}$) without TL. For probe 2 data, the ${\mathrm{ANN}}_{1}$ predicted MB concentration with RMSE=1.67  μM (MAPE=154.84%), whereas for probe 3 data, it predicted MB concentration with RMSE=4.1  μM (MAPE=361.08%), which is 2.45 times higher. Between probes 1 and 2, the SDS mismatch had RMSE=39.69  μm (MAPE=3.46%), whereas between probes 1 and 3, the SDS mismatch had RMSE=100.91  μm (MAPE=10.94%), which is 2.54 times higher. It is evident that a higher mismatch in SDS leads to higher prediction errors. Due to this greater mismatch between fiber positioning between probes 1 and 3 compared to that of probes 1 and 2, even after the application of TL the MB concentration recovery had somewhat higher RMSE=0.73  μM (MAPE=61.89%) for probe 3. This implies that there may be a maximum SDS difference that can be tolerated by this TL approach. Unfortunately, we do not have a sufficient selection of probes to test this directly, but this will be an area of future study.

Although our proposed TL algorithm has great potential in facilitating multicenter trials and ultimately widespread adaption of probe-based optical property recovery using ANN, it is not without limitations. We employed only three probes in this study, which prevents us from creating a general guideline involving tolerance in fiber positioning and its effect on optical property prediction. The differences among the probes were substantial but not overwhelmingly large. In future research, we are planning to investigate these issues more thoroughly by employing a larger number of probes with varying SDS. One weakness of the ANN approach is the inability to perform accurately and reliably outside the training range.^27^ Although the ANNs used in this study were trained on datasets that covered the range pertinent to our clinical application, there is a possibility that these ANNs will encounter out-of-domain data in a clinical scenario. We plan to utilize physics-guided neural networks for optical recovery in our future endeavors as these networks have shown superior out-of-domain performance for different tasks.^22^ The reliability of our TL algorithm may be compromised by uncertainties in the knowledge of the optical properties for the phantoms used for TL. This could be mitigated by the usage of solid phantoms with identical optical properties, including regular validation of these phantoms with another well-validated spectroscopy system.

## Supplementary Material



## Data Availability

Data are available upon reasonable request from the authors.
