# Supplementary material for: Application of transfer learning for rapid calibration of spatially resolved diffuse reflectance probes for extraction of tissue optical properties
Source: J Biomed Opt. 2024 Feb 28;29(2):027004. doi: 10.1117/1.JBO.29.2.027004 (PMC10901350; doi:10.1117/1.JBO.29.2.027004)
Supplement: Supplementary file 1 [file JBO_029_027004_SD001.pdf]

Supplemental Materials:

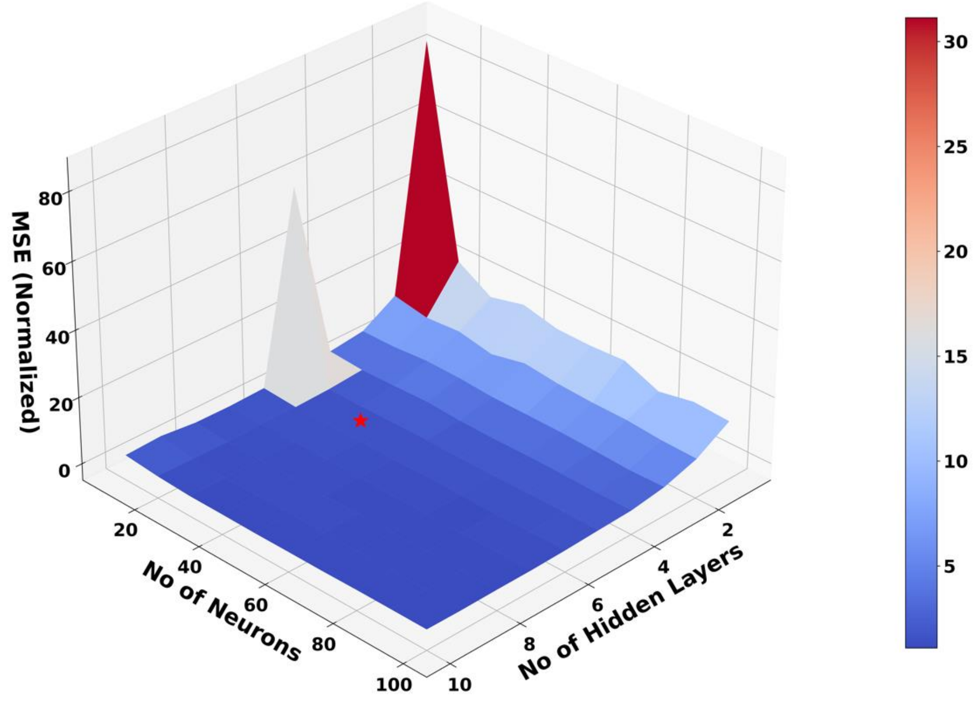

Figure S1: Surface plot showing the performance of different $ANN_{EXP1-MC1}$ network structures on a validation dataset. The number of hidden layers was varied from 1 to 10, and the number of neurons in each hidden layer was varied from 10 to 100 in an increment of 10, resulting in 100 different combinations. The mean squared error (MSE) for this validation set is a function of the number of hidden layers and the number of neurons in each hidden layer. The displayed MSE is normalized by the smallest MSE error of all combinations for ease of visualization. The selected network structure (5 hidden layers, 30 neurons in each hidden layer) is denoted with a red star.

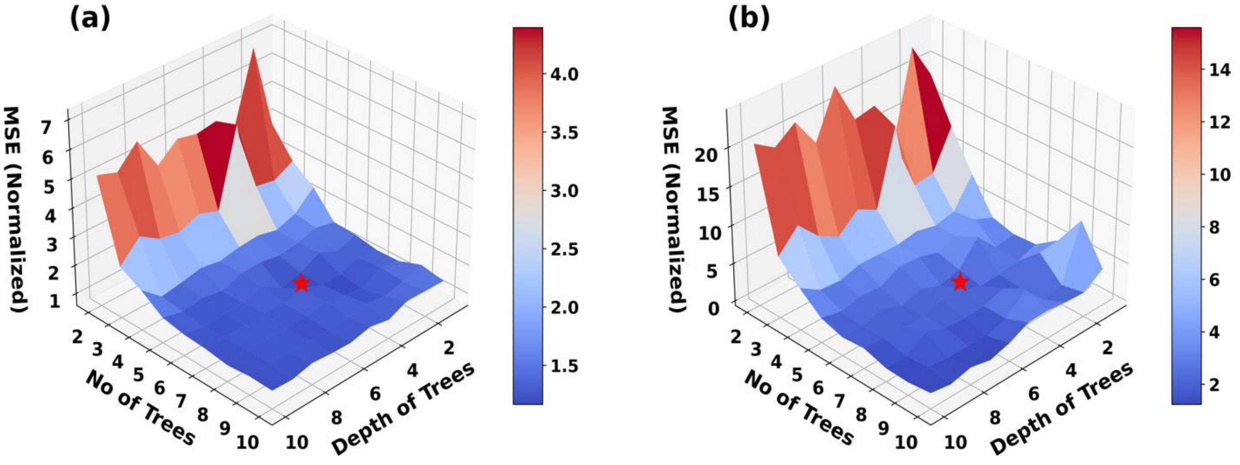

Figure S2: Surface plots showing the performance of different ANN_{MC1-OP} network structures on a validation dataset for (a) absorption coefficient and (b) reduced scattering coefficient. Both the number of trees and depth of trees were varied from 1 to 10, resulting in 100 different ANN models. The mean squared error (MSE) on this validation set is a function of the number of trees (number of ANNs) and depth of trees (number of layers). Displayed MSE for each is normalized by their respective smallest MSE for all combinations. The selected ANN_{MC1-OP} network (5 trees, depth of trees = 6) is denoted with a red star.

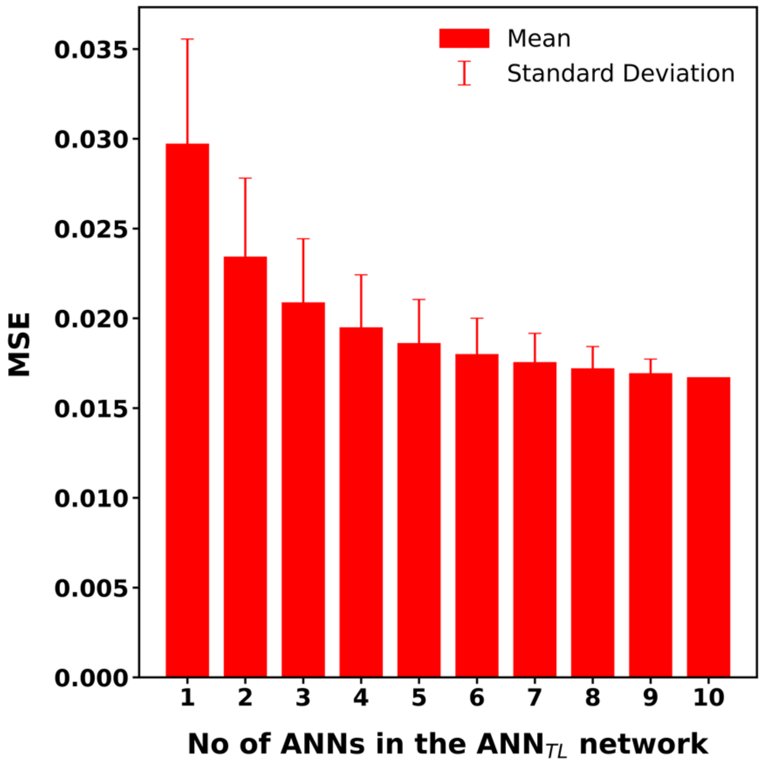

Figure S3: Prediction error for ANN$_{TL}$ networks with varying numbers of individual ANNs. The X-axis denotes the number of ANNs in the ANN$_{TL}$ network, and the Y-axis denotes the mean squared error (MSE) for that network. Solid bars represent mean values across all combinations of the specified number of ANNs, with error bars corresponding to standard deviation.
